# Inflammation resolution circuits are uncoupled in acute sepsis and correlate with clinical severity

**DOI:** 10.1172/jci.insight.148866

**Published:** 2021-08-09

**Authors:** Bakr Jundi, Do-Hyun Lee, Hyungkook Jeon, Melody G. Duvall, Julie Nijmeh, Raja-Elie E. Abdulnour, Mayra Pinilla-Vera, Rebecca M. Baron, Jongyoon Han, Joel Voldman, Bruce D. Levy

**Affiliations:** 1 Division of Pulmonary and Critical Care Medicine, Brigham and Women’s Hospital (BWH) and Harvard Medical School, Boston, Massachusetts, USA.; 2 Research Laboratory of Electronics, Massachusetts Institute of Technology, Boston, Massachusetts, USA.

**Keywords:** Inflammation, Pulmonology, Bacterial infections, Cellular immune response, Neutrophils

## Abstract

Sepsis is a critical illness characterized by dysregulated inflammatory responses lacking counter-regulation. Specialized proresolving mediators are agonists for antiinflammation and for promoting resolution, and they are protective in preclinical sepsis models. Here, in human sepsis, we mapped resolution circuits for the specialized proresolving mediators resolvin D1 and resolvin D2 in peripheral blood neutrophils and monocytes, their regulation of leukocyte activation and function ex vivo*,* and their relationships to measures of clinical severity. Neutrophils and monocytes were isolated from healthy subjects and patients with sepsis by inertial microfluidics and resolvin D1 and resolvin D2 receptor expression determined by flow cytometry. The impact of these resolvins on leukocyte activation was determined by isodielectric separation and leukocyte function by stimulated phagolysosome formation. Leukocyte proresolving receptor expression was significantly higher in sepsis. In nanomolar concentrations, resolvin D1 and resolvin D2 partially reversed sepsis-induced changes in leukocyte activation and function. Principal component analyses of leukocyte resolvin receptor expression and responses differentiated sepsis from health and were associated with measures of sepsis severity. These findings indicate that resolvin D1 and resolvin D2 signaling for antiinflammation and resolution are uncoupled from leukocyte activation in early sepsis and suggest that indicators of diminished resolution signaling correlate with clinical disease severity.

## Graphical abstract

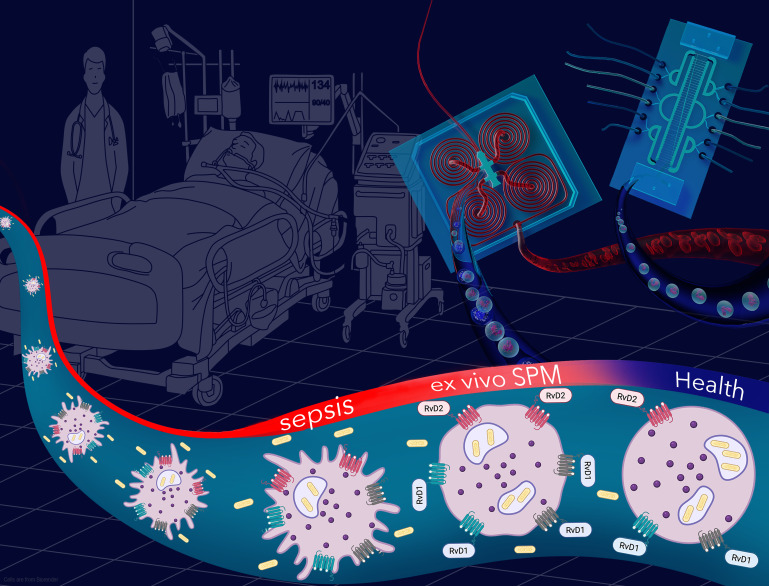

## Introduction

Sepsis commonly precipitates critical illness, is the leading cause of death from infection (1–4), and is characterized by an overexuberant and systemic immune response that can produce organ failure, shock, and death (4, 5). In sepsis, the uncontrolled inflammation can progress despite well-targeted antibiotics and adequate source control of the infection, suggesting that in many cases, the unfettered inflammatory response results from host rather than microbial factors (4, 6–8). Cytokines and lipid mediators are among the many proinflammatory mediators evoked in sepsis, but not all mediators provoke inflammation (6, 8–11). Resolution of inflammation is an active counter-regulatory process that is orchestrated in part by endogenous specialized proresolving mediators (SPMs) (10–12). In sepsis and other inflammatory diseases, leukocytes are bombarded by a diverse array of signals — including several that promote inflammation and others that promote resolution — and the leukocytes must interpret these signals to inform their functional responses (10, 12–16). We hypothesize that the state of leukocyte activation and their functional responses to ex vivo challenge reflects the in vivo clinical state in sepsis. If feasible in a clinical setting, these leukocyte measures have the potential to provide clinicians with earlier and more predictive and actionable information for a patient’s clinical course than the current routine critical care measures of total and differential leukocyte counting, surveillance for organ failure, or measures of physiological responses.

In microliter quantities of peripheral blood from patients with sepsis, polymorphonuclear neutrophil (PMN) dysfunction is apparent with significant decreases in degranulation, O_2_^–^ production, and phagocytosis; increased PMN activation is reflected in changes in the cells’ electrical properties (4, 7, 17–19). Sepsis can impact SPM production, yet the influence of sepsis on cellular resolution responses remains to be determined. D-series resolvins are a family of SPMs that display protective roles in preclinical sepsis models and are present in plasma from patients with sepsis (6, 8, 9, 13, 20, 21). Here, we have addressed our hypothesis by determining D-series resolvin receptor expression, the impact of resolvin D1 (RvD1) and RvD2 on leukocyte activation and function, and the relationship between these parameters of resolution and measures of sepsis clinical severity.

## Results

### Expression of SPM receptors DRV1, ALX, and DRV2 is increased on PMN subsets in sepsis.

Peripheral blood was collected from 8 healthy subjects and 18 patients with sepsis. The characteristics for healthy subjects and confirmed patients with sepsis are in Table 1.

**Figure 1:**
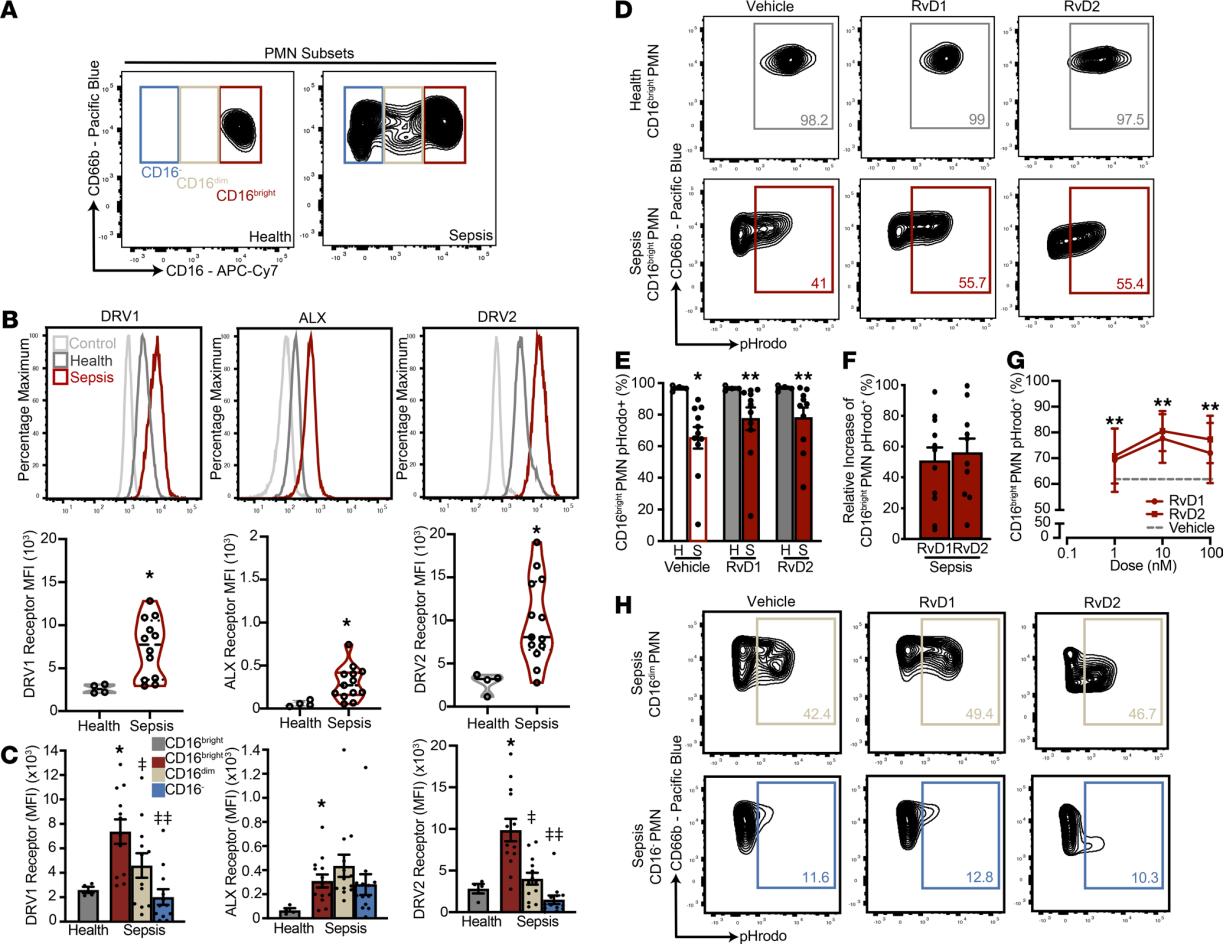
Upregulation of SPM receptors DRV1, ALX, and DRV2 on PMN in sepsis. PMN were isolated from 50 μL of peripheral blood using the spiral microfluidics system. (**A**) Flow cytometry contour plots identifying the various FSC^+^SSC^+^CD45^+^CD66b^+^PMN subsets based on CD16 surface expression (CD16^bright^, CD16^dim^, and CD16^–^) in isolated blood from healthy and sepsis subjects. (**B**) Representative flow cytometry histogram plots (upper panels) and violin graphs (lower panels) of CD16^bright^ PMN (FSC^+^SSC^+^CD45^+^CD66b^+^CD16) surface receptor expression of DRV1, ALX, and DRV2 in control (fluorescence minus 1, light gray), health (dark gray), and sepsis (crimson). Health, *n* = 4; Sepsis, *n* = 12–13. (**C**) Mean fluorescence intensity (MFI) of surface receptor expression of DRV1, ALX, and DRV2 in all PMN subsets in sepsis and health. Health, *n* = 4; Sepsis, *n* = 12–13. (**D** and **E**) Representative flow cytometry contour plots and frequency of pHrodo^+^CD16^bright^ PMN in sepsis (crimson) and health (dark gray) after incubation with exogenous RvD1 (100 nM), RvD2 (100 nM), or vehicle (<0.01% v/v EtOH) for 15 minutes at 37°C. Health, *n* = 4; Sepsis, *n* = 11. (**F**) The relative increase of pHrodo^+^CD16^bright^ PMN with exogenous RvD1 (100 nM) and RvD2 (100 nM) in sepsis calculated as (SPM sepsis – vehicle sepsis)/(vehicle healthy – vehicle sepsis). Sepsis, *n* = 11. (**G**) Concentration-response curve of the frequency of pHrodo^+^CD16^bright^ PMN to varying concentrations of RvD1 (circle, crimson), RvD2 (square, crimson), or vehicle (mean value, dashed gray line). Sepsis, *n* = 6. (**H**) Representative flow cytometry contour plots of pHrodo^+^ CD16^dim^ and CD16^–^ PMN with exogenous RvD1 (100 nM), RvD2 (100 nM), and vehicle (<0.01% v/v EtOH), *n* = 11. Values are expressed as the mean ± SEM. *P < 0.05 for sepsis versus health by unpaired, 2-tailed *t* test; **P < 0.05 for vehicle versus RvD1 or RvD2 by paired, 2-tailed *t* test; ^‡^*P* < 0.05 for CD16^dim^ versus CD16^bright^ PMN by paired, 2-tailed *t* test; ^‡‡^*P* < 0.05 CD16^–^ versus CD16^bright^ PMN by paired, 2-tailed *t* test. H, Health. S, Sepsis.

To investigate counter-regulatory proresolving mechanisms in sepsis, we first determined PMN expression level of the 2 RvD1 receptors, DRV1and ALX, and the RvD2 receptor, DRV2. After isolation by inertial microfluidic separation, flow cytometry uncovered mature CD16^bright^ PMN (FSC^+^SSC^+^CD45^+^CD66b^+^CD16^+^), immature CD16^dim^, and CD16^–^ PMN in peripheral blood from patients with sepsis drawn within 72 hours of presentation (day 0), whereas only mature CD16^bright^ PMN were detected in healthy blood (Figure 1A; gating strategy in Supplemental Figure 1; supplemental material available online with this article; https://doi.org/10.1172/jci.insight.148866DS1). DRV1, ALX, and DRV2 were all expressed on healthy and sepsis CD16^bright^ PMN, with mean fluorescent intensity (MFI) significantly increased in patients with sepsis by ~3-fold for DRV1, 5-fold for ALX, and 4-fold for DRV2 compared with healthy subjects (Figure 1B). While mature and immature PMN expressed DRV1, ALX, and DRV2 in sepsis, the MFI for DRV1 and DRV2 on CD16^bright^ PMN was significantly higher than on CD16^dim^ and CD16^–^ PMN (Figure 1C). There was no significant change in DRV1, ALX, and DRV2 levels on all PMN subsets on days 3 and 7 (Supplemental Figure 2A).

### RvD1 and RvD2 partially reversed the diminished PMN phagolysosome activity in sepsis.

In sepsis, PMN functional responses are decreased (4, 7, 17, 22). Given the increased expression of DRV1 and DRV2 (Figure 1B), we determined the impact of exogenous SPM ligands for these receptors on PMN function. Isolated PMN were exposed to RvD1 or RvD2 (100 nM, 15 minutes) before incubation with pHrodo red *E*. *coli* bioparticles (15 minutes) to detect phagolysosome formation (Supplemental Figure 2B and Figure 1D) (7). PMN from patients with sepsis (vehicle control) had significantly lower numbers of pHrodo^+^CD16^bright^ PMN than cells from healthy subjects (Figure 1, D and E), indicating diminished PMN function in early sepsis. In the presence of exogenous RvD1 or RvD2, PMN from patients with sepsis displayed a significant increase in the mean percentage of pHrodo^+^CD16^bright^ PMN (Figure 1, D and E). By day 3, the percentage of pHrodo^+^CD16^bright^ PMN had increased in septic patients to nearly 100%, and no further increase could be evaluated for RvD1 or RvD2 (Supplemental Figure 2C). The partial reversal of PMN functional defects in sepsis by RvD1 or RvD2 relative to vehicle was calculated as a mean relative increase of pHrodo^+^CD16^bright^ PMN (SPM sepsis – vehicle sepsis)/(vehicle healthy – vehicle sepsis), and RvD1 and RvD2 gave relative increases of 50.7% ± 9.0% and 56.1% ± 9.4% (mean ± SEM), respectively (Figure 1F). For reference, the mean absolute increase of pHrodo^+^CD16^bright^ PMN in sepsis, which was calculated by (SPM sepsis – vehicle sepsis) was 11.3% ± 2.0% with RvD1 and 15.3% ± 2.3% with RvD2 (mean ± SEM) (Supplemental Figure 2D). Concentration dependency was assessed by adding RvD1 and RvD2 at 1, 10, and 100 nM to PMN from patients with sepsis. A significant increase in the mean percentage of pHrodo^+^CD16^bright^ PMN was present at all RvD1 and RvD2 nanomolar concentrations relative to vehicle (Figure 1G).

Given the differential receptor expression on mature and immature PMN subsets in sepsis, we looked at the phagocytic capacity of those subsets and the cell responses to exogenous RvD1 and RvD2. The mean absolute increases in CD16^dim^ PMN phagocytic capacity following RvD1 and RvD2 exposure were lower than those observed in mature CD16^bright^ PMN (6.2% ± 1.8% and 11.6% ± 3.0% [mean ± SEM], respectively) and were even less in CD16^–^ PMN (Figure 1H and Supplemental Figure 2D). Distinct from mature PMN, significant relative increases with RvD1 and RvD2 could not be determined for CD16^dim^ and CD16^–^ PMN, as there were too few immature PMN in healthy samples to make the calculations.

### RvD1 and RvD2 limit PMN activation in sepsis and healthy individuals.

**Figure 2:**
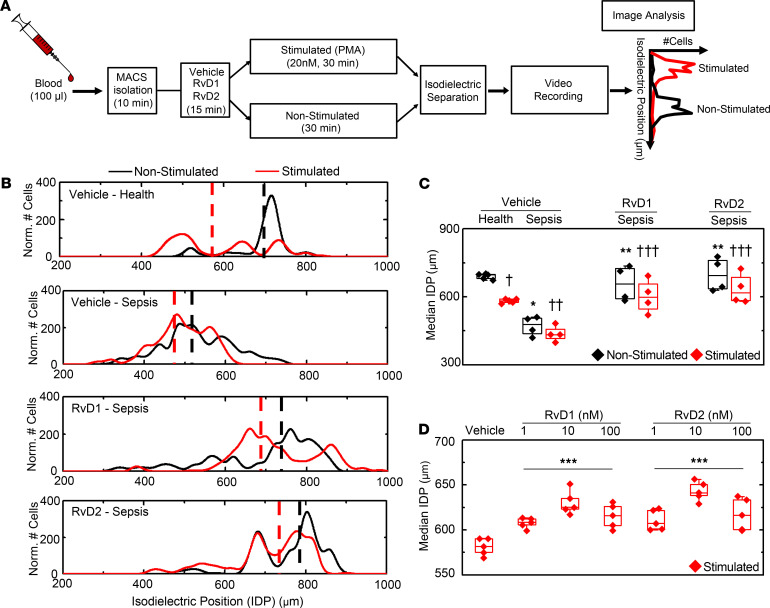
RvD1 and RvD2 limit PMN activation by IDS in sepsis and healthy individuals. (**A**) Experimental workflow for the isolation and assessment of PMN activation using isodielectric separation (IDS) to determine their electrical signature, isodielectric position (IDP). After isolation, PMN were first incubated with vehicle (<0.01% v/v EtOH), RvD1 (100 nM), or RvD2 (100 nM) for 15 minutes; they were then either stimulated by PMA (20 nM, red) or nonstimulated (black) for 30 minutes. Their IDPs were measured at 7 MHz frequency voltage. (**B**) Histogram plots of IDP distributions representative of healthy subjects (top panel) and patients with sepsis (bottom 3 panels). The dashed lines on the histogram plots are the median IDP, and their shift is determined upon stimulation of cells with PMA (red lines). (**C**) Box and whisker plots (median, 25th, and 75th percentiles) of the median IDP in nonstimulated (black) or PMA-stimulated (red) PMN of healthy subjects and patients with sepsis. (**D**) Box and whisker plots (median, 25th, and 75th percentiles) of the concentration-response curve of the median IDP of PMA-activated PMN from healthy subjects with exogenous RvD1 and RvD2. †*P* < 0.05 for nonstimulated veh versus stimulated veh in healthy subjects by paired, 2-tailed *t* test. *P < 0.05 for nonstimulated veh-health versus nonstimulated veh-sepsis by unpaired, 2-tailed *t* test. ††*P* < 0.05 for stimulated veh-health versus stimulated veh-sepsis by unpaired, 2-tailed *t* test. **P < 0.05 for unstimulated RvD1 versus nonstimulated veh in sepsis by unpaired, 2-tailed *t* test. ^†††^*P* < 0.05 for stimulated RvD1 and RvD2 versus nonstimulated vehicle in health or sepsis by paired, 2-tailed *t* test. ***P < 0.05 for concentration-response curve of median IDP of stimulated PMN from healthy donors with exogenous RvD1, RvD2, and vehicle by 1-way ANOVA. *n* = 5 healthy subjects, *n* = 4 sepsis.

To determine the impact of SPM on leukocyte activation in sepsis, the isodielectric position (IDP) of freshly isolated PMN was determined after incubation with vehicle (<0.01% v/v EtOH), RvD1, or RvD2 (both 100 nM) followed by PMA (20 nM, stimulated) or vehicle (nonstimulated) (Figure 2A). PMN from healthy subjects stimulated with PMA significantly lowered the median IDP, at 7 MHz frequency, to 581 ± 4.10 μm (mean ± SEM, red dashed line) relative to nonstimulated PMN at 690 ± 5.63 μm (mean ± SEM, black dashed line) (Figure 2, B and C). Of interest, PMN from patients with sepsis had similar median IDP with PMA (436 ± 17.15 μm, mean ± SEM, red dashed line) as nonstimulated PMN (471 ± 21.38 μm, mean ± SEM, black dashed line) (Figure 2, B and C), indicating that PMN isolated from patients with sepsis were activated prior to isodielectric separation (IDS). Consistently, PMN from patients with sepsis had a significantly lower median IDP than PMN from healthy subjects before and after PMA stimulation (Figure 2C).

When PMN from healthy subjects were exposed to the SPMs RvD1 and RvD2 (100 nM, 15 minutes), nonstimulated cells had a median IDP of 654 ± 8.36 μm (RvD1) and 661 ± 8.02 μm (RvD2) (mean ± SEM) (Supplemental Figure 3, A and B). Preincubation of PMN from healthy subjects with RvD1 or RvD2 blunted the change in IDP with PMA relative to vehicle; the median IDP for PMA-stimulated PMN was 615 ± 6.00 μm (RvD1) and 617 ± 7.95 μm (RvD2) (mean ± SEM) (Supplemental Figure 3, A and B). In patients with sepsis, preincubation of PMN with RvD1 or RvD2 gave a median IDP at similar levels as PMN from healthy subjects (black dashed line) and prevented significant PMA-initiated shifts in IDP (red dashed line) (Figure 2, B and C).

With marked changes in IDP with SPM compared with vehicle control, the differences between the median IDP of PMN stimulated with PMA or vehicle was calculated as a Δ median IDP (Δ_med_IDP = median IDP_veh_ − median IDP_PMA_). PMN (with vehicle) from healthy subjects had a Δ_med_IDP of 109 ± 2.92 μm (mean ± SEM), whereas SPM exposure led to a significant decrease in the Δ_med_IDP to 39 ± 4.20 μm (RvD1) and 45.5 ± 2.16 μm (RvD2) (mean ± SEM) (Supplemental Figure 3C). To determine whether these changes were dependent upon concentration, we assessed the actions of 1, 10, and 100 nM RvD1 or RvD2 on IDS responses. In PMA-stimulated PMN, compared with nonstimulated control, the SPMs gave a significant increase in the median IDP at all concentrations (red, Figure 2D). In the absence of PMA, there were significant SPM-mediated decreases in median IDP at all concentrations relative to vehicle (black, Supplemental Figure 3D). A corresponding significant decrease in Δ_med_IDP was present at all SPM concentrations relative to vehicle (Supplemental Figure 3E).

### Expression of SPM receptors DRV1, ALX, and DRV2 is increased on monocyte subsets in sepsis.

**Figure 3:**
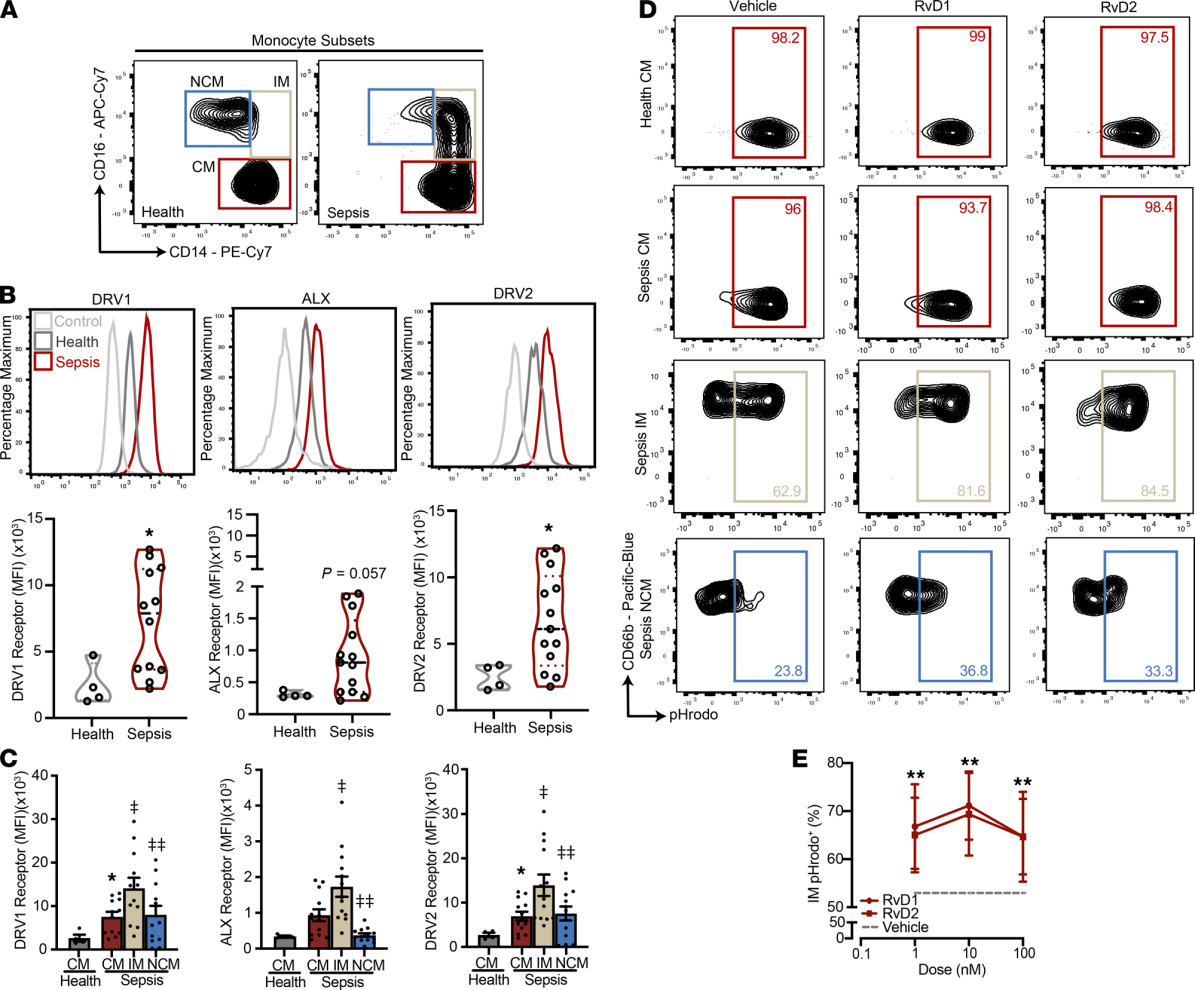
Upregulation of SPM receptors DRV1, ALX, and DRV2 in monocytes in sepsis. Monocytes were isolated from were isolated from 50 μL of peripheral blood using the closed-loop operation of spiral microfluidics system. (**A**) Flow cytometry contour plots identifying the various FSC^+^SSC^–^CD45^+^CD66b^–^ monocyte subsets based on CD16 and CD14 surface expression (classical, intermediate, and nonclassical monocytes) in isolated blood from healthy subjects and patients with sepsis. Classical monocytes were defined as FSC^+^SSC^–^CD45^+^CD66b^–^CD16^lo^CD14^hi^, intermediate monocytes as FSC^+^SSC^–^CD45^+^CD66b^–^CD16^hi^CD14^hi^, and nonclassical monocytes as FSC^+^SSC^–^CD45^+^CD66b^–^CD16^hi^CD14^lo^. (**B**) Representative flow cytometry histogram plots (upper panel) and violin graphs (lower panel) of classical monocytes (CM) surface receptor expression of DRV1, ALX, and DRV2 in control (fluorescence minus 1, light gray), health (dark gray), and sepsis (crimson). Health, *n* = 4; Sepsis, *n* = 12–13. (**C**) Mean fluorescence intensity (MFI) of surface receptor expression of DRV1, ALX, and DRV2 among all monocyte subsets: classical monocytes (CM, crimson), intermediate monocytes (IM, beige), and nonclassical monocytes (NCM, blue) in health (*n* = 4) and sepsis (*n* = 12–13). (**D**) Representative flow cytometry contour plots of pHrodo^+^ classical (CM, crimson), intermediate (IM, beige), and nonclassical (NCM, blue) monocytes from patients with sepsis incubated with vehicle (<0.01% EtOH), RvD1 (100 nM), or RvD2 (100 nM) for 15 minutes at 37°C, *n* = 10–11. (**E**) Concentration-response curve of the frequency of pHrodo^+^ intermediate monocytes varying concentrations of RvD1 (circle, crimson), RvD2 (square, crimson), or vehicle (mean value, dashed gray line). Sepsis, *n* = 6. Values are expressed as the mean ± SEM. *P < 0.05 for sepsis versus health by unpaired, 2-tailed *t* test; **P < 0.05 for vehicle versus RvD1 or RvD2 by paired, 2-tailed *t* test; ^‡^*P* < 0.05 for IM versus CM by paired, 2-tailed *t* test; ^‡‡^*P* < 0.05 NCM versus IM by paired, 2-tailed *t* test.

Classical monocytes (FSC^+^SSC^–^CD45^+^CD66b^–^CD16^lo^CD14^hi^) were isolated from peripheral blood using inertial microfluidics (Supplemental Figure 1 and Figure 3A), and surface expression of DRV1, ALX, and DRV2 receptors was determined for cells from healthy subjects (dark gray) and patients with sepsis (Figure 3B). Similar to CD16^bright^ PMN (Figure 1), classical monocyte expression of these SPM receptors was significantly increased by ~3-fold in sepsis relative to health (Figure 3B). In addition to classical monocytes, intermediate and nonclassical monocytes were present in peripheral blood from patients with sepsis (Figure 3A). Of interest, DRV1, ALX, and DRV2 expression on intermediate monocytes in sepsis was significantly higher at presentation (day 0) relative to classical and nonclassical monocytes (Figure 3C). No significant changes in SPM receptor expression were observed on the monocyte subsets on days 3 and 7 (Supplemental Figure 4A).

### RvD1 and RvD2 increase phagolysosome activity in select monocyte subsets in sepsis.

Given the differential expression of SPM receptors on monocyte subsets with sepsis, the impact of exogenous RvD1 and RvD2 (100 nM) on phagolysosome activity was determined (see Methods). Nearly all classical monocytes from patients with sepsis and healthy subjects were pHrodo^+^ and without significant differences (Figure 3D and Supplemental Figure 4B). With exogenous RvD1 and RvD2, there were no significant changes in the already high percentage of pHrodo^+^ classical monocytes (Figure 3D and Supplemental Figure 4B). The mean absolute increases in the percentage of pHrodo^+^ classical monocytes (described above) were 4.3 ± 1.3 (RvD1) and 4.4 ± 1.4 (RvD2) (mean ± SEM) (Supplemental Figure 4C). In addition, there were no significant changes in phagocytic capacity of classical monocytes with SPMs between 1 and 100 nM of RvD1 and RvD2 (Supplemental Figure 4D). Significant differences with SPM were observed with intermediate monocytes from patients with sepsis, with the mean absolute increases in intermediate monocyte phagocytic capacity at 10.9 ± 2.5 (RvD1) and 12.6 ± 2.5 (RvD2) (mean ± SEM) (Supplemental Figure 4C). It is important to note that intermediate and nonclassical monocytes were not present in sufficient numbers for analysis in healthy subjects. These increases with intermediate monocytes were higher than those seen with classical monocytes from the same subjects (Supplemental Figure 4C). The mean absolute increases in nonclassical monocyte phagocytic capacity were 7.5 ± 1.9 (RvD1) and 5.6 ± 1.8 (RvD2) (mean ± SEM); these were lower than seen with the intermediate monocytes (Supplemental Figure 4C). The increased phagocytic capacity of intermediate monocytes with SPMs was concentration dependent between 1 and 100 nM of RvD1 (circle, crimson) and RvD2 (square, crimson), with significant increases in the mean percentage of pHrodo^+^ intermediate monocytes at all concentrations relative to vehicle (dashed gray line) (Figure 3E).

### Relationships between DRV1, ALX, and DRV2 expression and leukocyte functional responses to SPM in sepsis.

**Figure 4:**
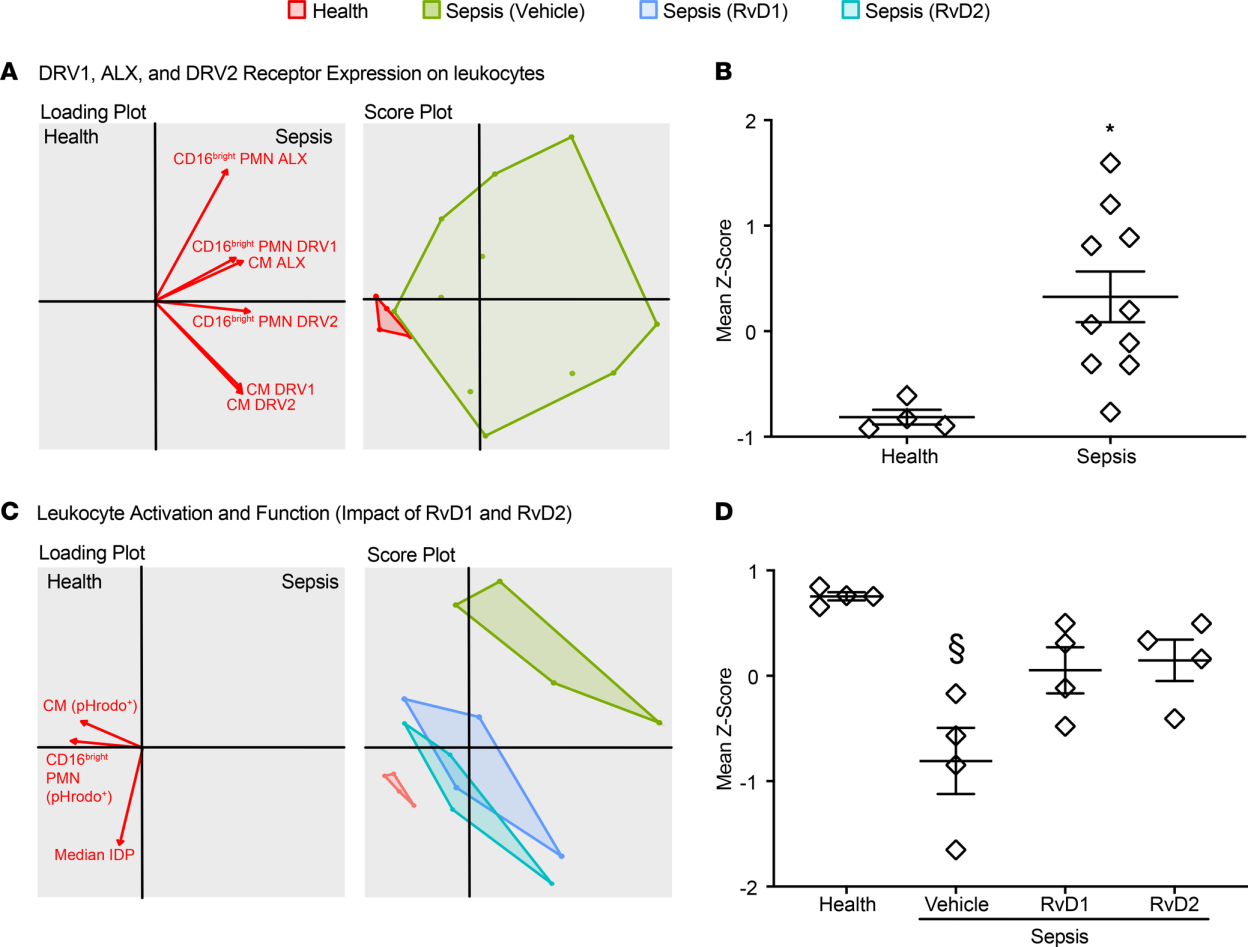
Differential expression of DRV1, ALX, and DRV2 receptors and functional responses in sepsis are counter regulated by RvD1 and RvD2. (**A**–**D**) Two-dimensional score and loading plots from multivariate principal component analysis (**A **and** C**) and mean *Z* score (**B **and** D**) were performed for levels of expression of DRV1, ALX, and DRV2 on leukocytes, and leukocyte activation and function, as indicated by median IDP and percentage of pHrodo^+^CD16^bright^ PMN, and pHrodo^+^ classical monocytes (CM). Leukocytes from healthy subjects are indicated in red, those from patients with sepsis incubated with vehicle control are in green, and addition of RvD1 and RvD2 is indicated in blue and aquamarine, respectively. The mean *Z* score was derived from individual variable *Z* scores in a given subject (*n* = 4, healthy subjects; *n* = 10, patients with sepsis in **A** and **B**; and *n* = 4, patients with sepsis in **C** and **D**). The data presented include only subjects with complete data set of all variables (receptor, function, and activation). Values are expressed as the mean ± SEM. *P < 0.05 health versus sepsis by unpaired, 2-tailed *t* test. ^§^*P* < 0.05 Kruskal-Wallis test, followed by Dunn’s test for multiple comparisons.

DRV1, ALX, and DRV2 expression on leukocytes differentiated patients with sepsis at presentation from healthy subjects as demonstrated by the score plot (Figure 4A) and aggregated standard scores (*Z* scores) (Figure 4B). In addition, leukocyte activation and function (median IDP, pHrodo^+^CD16^bright^ PMN, and pHrodo^+^ classical monocytes) differentiated sepsis from health (Figure 4, C and D). The changes in leukocytes from patients with sepsis in their phagocytic responses with exogenous RvD1 and RvD2 returned the score plot and aggregated *Z* scores toward those of healthy subjects (Figure 4, C and D). Moreover, the aggregated *Z* scores of PMN Δ_med_IDP also distinguished sepsis from health, and SPM exposure significantly shifted values of cells from patients with sepsis back toward those from healthy subjects (Supplemental Figure 5, A and B).

### Relationships between RvD1 signaling circuits and disease severity in sepsis for PMN and intermediate monocytes.

**Figure 5:**
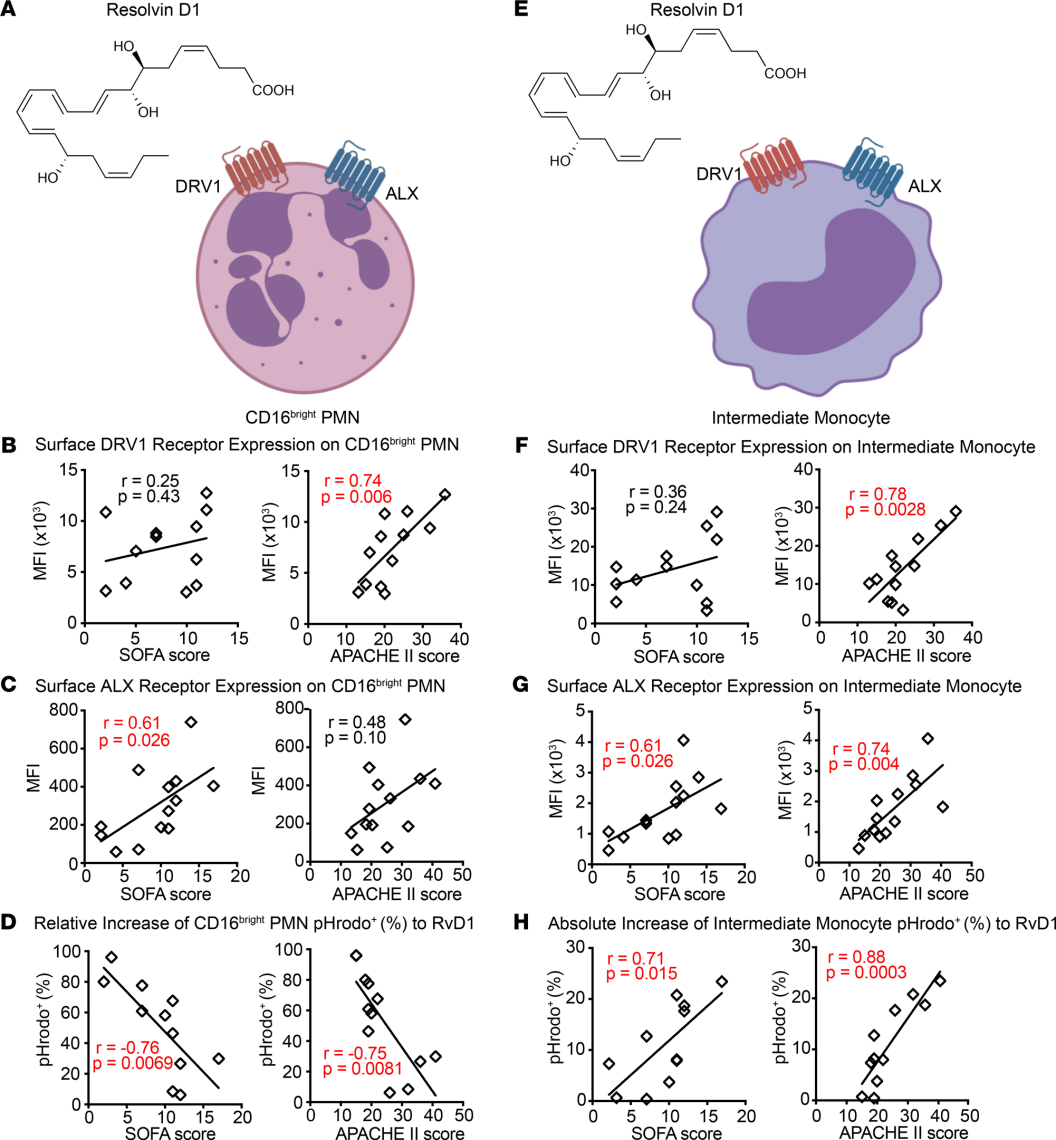
Correlation between leukocyte responses to RvD1 and sepsis clinical severity. (**A**) Schematic diagram of the chemical structure of RvD1 and its receptors (DRV1 and ALX) on the surface of CD16^bright^ PMN. (**B**–**D**) The correlation between clinical severity indicators (SOFA and APACHE II) (other severity indicators such as status of mechanical ventilation and mortality are included in the included in Supplemental Figure 6) and expression levels of surface receptors DRV1 (*n* = 12) (**B**) and ALX (*n* = 13) (**C**), and relative increase of frequency of pHrodo^+^ CD16^bright^ PMN with exogenous RvD1 (*n* = 11) (**D**), was determined. (**E**) Schematic diagram of the chemical structure of RvD1 and its receptors (DRV1 and ALX) on the surface of intermediate monocytes. (**F**–**H**) The correlation between clinical severity indicators (SOFA and APACHE II) (other severity indicators such as status of mechanical ventilation and mortality are included in the Supplemental Figures), and expression levels of surface receptors DRV1 (*n* = 12) (**F**), and ALX (*n* = 13) (**G**), and absolute increase of frequency of pHrodo^+^ intermediate monocyte with exogenous RvD1 (*n* = 11) (**H**), was determined. The Pearson correlation *r* value and significance are noted; red indicates significance of *P* < 0.05 and regression lines are shown. Cell images are from Biorender.

Next, individual SPM signaling circuits were examined, first for expression of RvD1 receptors (DRV1 and ALX) and parameters of leukocyte responses to RvD1 in order to determine if there was a relationship to sepsis clinical severity (Figure 5, A and E). Four measures of disease severity were used in these analyses — namely, the sequential organ failure assessment (SOFA) score, acute physiologic assessment and chronic health evaluation (APACHE) II score, status of mechanical ventilation (on/off), and mortality. CD16^bright^ PMN DRV1 expression positively correlated with APACHE II score, and ALX expression positively correlated with SOFA score (Figure 5, B and C). In addition, the relative increase of pHrodo^+^CD16^bright^ PMN following incubation with exogenous RvD1 inversely correlated to SOFA and APACHE II scores (Figure 5D). The RvD1-enhanced phagocytosis of bacterial particles by PMN was associated with lower SOFA and APACHE II scores, and it was also associated with no requirement for mechanical ventilation and with survival (Figure 5D and Supplemental Figure 6A). Intermediate monocyte DRV1 and ALX expression positively correlated with APACHE II score, and ALX also positively correlated with SOFA score (Figure 5, F and G). In sharp contrast to PMN, there was a positive correlation with RvD1-enhanced phagocytosis of bacterial particles by intermediate monocytes and SOFA and APACHE II scores (Figure 5H), and there was a trend for increased frequency of mechanical ventilation and death (Supplemental Figure 6B), suggesting a distinct pathogenic role for intermediate monocytes in sepsis. No significant correlations were seen with other disease severity indicators (Supplemental Figure 6, C–G).

### Relationships between RvD2 signaling circuits and disease severity in sepsis for PMN and intermediate monocytes.

**Figure 6:**
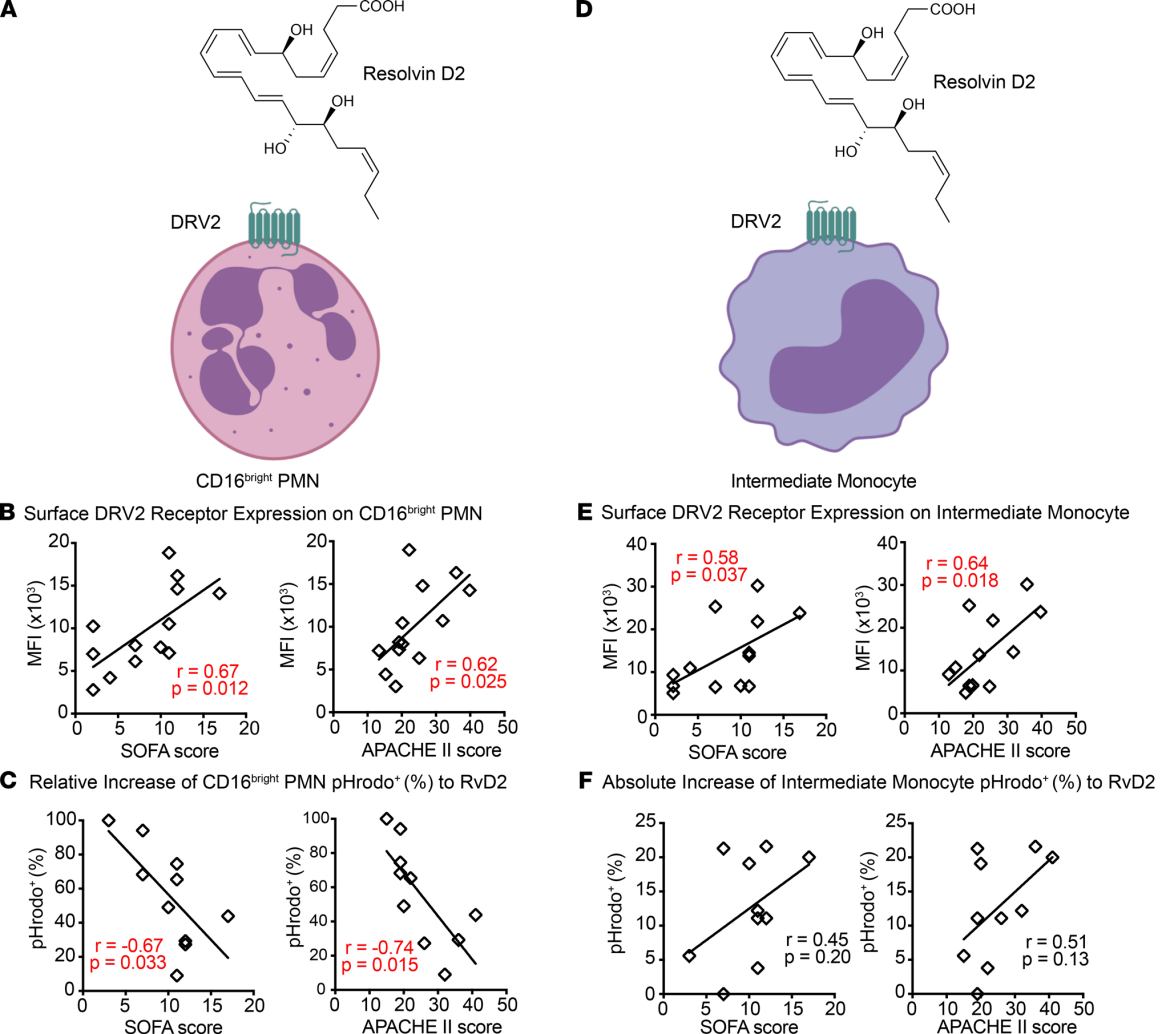
Correlation between leukocyte responses to RvD2 and sepsis clinical severity. (**A**) Schematic diagram of the chemical structure of RvD2 and its receptor (DRV2) on the surface of CD16^bright^ PMN. (**B** and **C**) The correlation between clinical severity indicators (SOFA and APACHE II) (other severity indicators such as status of mechanical ventilation and mortality are included in the Supplemental Figures) and expression levels of surface receptor DRV2 (*n* = 13) (**B**), and relative increase of frequency of pHrodo^+^ CD16^bright^ PMN with exogenous RvD2 (*n* = 10) (**C**), was determined. (**D**) Schematic diagram of the chemical structure of RvD2 and its receptor (DRV2) expressed on the surface of intermediate monocyte. (**E** and **F**) The correlation between clinical severity indicators (SOFA, APACHE II) (other severity indicators such as status of mechanical ventilation and mortality are included in the Supplemental Figures), and expression levels of surface receptor DRV2 (*n* = 13) (**E**), and absolute increase of frequency of pHrodo^+^ Intermediate monocyte with exogenous RvD2 (*n* = 10) (**F**), was determined. The Pearson correlation *r* value and significance are noted; red indicates significance of *P* < 0.05 and regression lines are shown. Cell images are from Biorender.

Expression of the RvD2 receptor, DRV2, and parameters of leukocyte responses to RvD2 were next determined to examine potential relationships to sepsis clinical severity (Figure 6A). CD16^bright^ PMN DRV2 expression positively correlated SOFA and APACHE II scores (Figure 6B). Similar to RvD1 (Figure 5), the relative increases in pHrodo^+^CD16^bright^ PMN with RvD2 gave significant inverse correlations to SOFA and APACHE II scores (Figure 6C). Higher responses to RvD2-enhanced phagocytosis of bacterial particles were associated with lower SOFA and APACHE II scores (Figure 6C) and had trends for not requiring mechanical ventilation and survival (Supplemental Figure 7A). Intermediate monocyte DRV2 expression positively correlated with SOFA and APACHE II scores (Figure 6, D and E). While not reaching statistical significance, similar to RvD1, the trends for RvD2-mediated increases in pHrodo^+^ intermediate monocytes were for a positive correlation with SOFA and APACHE II scores (Figure 6F). No significant correlations were seen with other disease severity indicators (Supplemental Figure 7, B–E).

## Discussion

Here, we have identified several changes in leukocyte resolution signaling circuits in early sepsis that, together, would unleash acute inflammation by disrupting counter-regulatory restraint mechanisms. Closed-loop inertial microfluidics was used for label-free isolation of leukocytes from microliter amounts of peripheral blood for immunophenotyping and functional analysis (as in ref. 7). In patients with sepsis, PMN activation and host protective responses — namely, phagocytosis of bacterial particles — was impaired. Of interest, leukocyte SPM receptor expression was higher in sepsis than health, and DRV1, ALX, and DRV2 expression correlated with several parameters of disease severity. Administration of SPMs ex vivo partially corrected the leukocyte defects in a cell type–specific manner that was associated with SPM receptor expression. Endogenous leukocyte activation in sepsis was detectable by IDS, and PMN activation was reversed ex vivo by SPMs in low nanomolar amounts in a concentration-dependent manner. In principal component analyses (PCA), measures of leukocyte SPM receptor expression, function, and activation differentiated sepsis from health, and ex vivo SPM-mediated proresolving responses evolved the sepsis PMN measures toward those from healthy subjects. It was also notable that SPM-enhanced phagocytosis by PMN and intermediate monocytes was directionally similar yet were in sharp contrast when correlated with measures of sepsis clinical severity. Together, these findings have uncovered marked changes in leukocyte activation and cell type–specific resolution signaling in sepsis and provide several potential cellular and molecular biomarkers for clinical correlation with critical illness and its severity.

Inflammation resolution requires regulation of PMN activation, control of further PMN infiltration into tissues, and promotion of cellular clearance mechanisms such as macrophage-mediated phagocytosis of microbes, cellular debris, and apoptotic cells (4, 6, 10, 12). RvD1 and RvD2 are SPMs that interact with specific receptors at high affinity (*K_D_* in low nM) to transduce their actions (11–13, 20, 21, 23–25). Mature CD16^bright^ PMN expressed RvD1 receptors (DRV1, ALX) and DRV2, all of which were increased with sepsis. Earlier stage CD16^dim^ and CD16^–^ PMN also expressed these SPM receptors, and there was a gradient of DRV1 and DRV2 expression that correlated with PMN maturation, suggesting a role for these SPM receptors in PMN ontogeny. SPMs have been identified in BM (26), and these and other lipid mediators have been postulated to play important regulatory functions in myelopoiesis (reviewed in ref. 27). Peripheral blood monocytes also expressed elevated levels of these SPM receptors in sepsis. Of particular note was the expansion of the intermediate monocytes in sepsis, as these specialized monocytes provoke inflammation and are linked to sepsis and its clinical severity (4, 7, 28–30). Here, the intermediate monocytes had the highest SPM receptor expression among monocytes, supporting the notion that these proinflammatory cells are also responsive to environmental proresolving cues. Mechanisms that increased leukocyte expression of DRV1 and DRV2 in sepsis were not established here but may relate to either resolvin availability or select proinflammatory mediators (e.g., GM-CSF) (31, 32).

Current profiling of clinical sepsis characteristics is primarily limited to leukocyte total and differential counts by complete blood count (CBC) (1–3, 5, 7, 33–36). These measures are devoid of leukocyte activation parameters that might better reflect functional leukocyte responses. Building off the concept that leukocytes are active integrators of hundreds of diverse environmental signals, which together direct functional responses, we employed IDS as a direct and sensitive measure of leukocyte activation. PMN median IDP and Δ_med_IDP is lower in sepsis than in health and correlates with SOFA scores for sepsis clinical severity (7). Here, we confirmed that leukocyte activation during sepsis influenced the PMN median IDP and Δ_med_IDP — relationships that correlated with sepsis clinical severity. In this study, we have uncovered a potent (low nM) action for RvD1 and RvD2 to dampen cell activation in freshly isolated PMN from patients with sepsis and to inhibit cell activation by the potent soluble, nonreceptor dependent stimulus PMA. To this end, the change in PMN IDP with PMA was significantly blunted, with the SPMs leading to a markedly reduced Δ_med_IDP. These findings with exogenous SPM suggest a relative functional deficiency in SPMs in vivo during sepsis. Of note, RvD1 and RvD2 have been detected in plasma in sepsis, with levels associated with survival (9). Together, these results indicate that microliter sampling of peripheral blood by IDS can uncover the endogenous activation state of leukocytes that is reflective of sepsis clinical severity, and they suggest that IDS has the potential to serve as a predictive clinical biomarker for critical illness.

Direct relationships for individual SPM signaling circuits and sepsis severity uncovered positive correlations between leukocyte DRV1 and DRV2 expression and measures of sepsis severity. In view of these positive correlations, responses to exogenous SPM were examined and increased ex vivo RvD1- and RvD2-induced PMN phagocytic responses to bacterial challenge. Increased pHrodo-labeled particles in phagocytes may be secondary to either increased phagocytosis or increased phagolysosome acidification, so the increases observed with SPMs could have resulted from either or both of these cellular mechanisms. Of note, the SPM-evoked increases in neutrophil phagocytic responses were associated with lower severity scores, supporting a protective function for these resolution circuits in sepsis PMN responses that are apparently uncoupled in vivo. In sharp contrast, increased SPM responsiveness for intermediate monocyte phagocytosis of bacterial particles correlated with worse outcomes. These findings underscore the cell type–specific responses of PMN and intermediate monocytes to SPMs, and they highlight the provocative role for intermediate monocytes in the dysregulated immune responses in sepsis. Recent studies have identified these monocyte subsets (7), which can be even further defined in molecular terms (29), as central to sepsis pathophysiology.

In summary, in this basic experimental study in humans, peripheral blood leukocytes in sepsis were differentiated from health by several measures. In addition to CD16^bright^ PMN and classical monocytes, CD16^dim^ and CD16^–^ PMN numbers, as well as intermediate and nonclassical monocyte numbers, increased in patients with sepsis. DRV1, ALX, and DRV2 receptor expression was increased in early sepsis — in particular, on CD16^bright^ PMN that had defects in phagocytosis of bacterial particles despite evidence for aberrant electrical properties, suggestive of cell activation. Ex vivo exposure to low nM concentrations of the SPMs, RvD1, and RvD2 elicited cell responses consistent with intact resolution signaling circuits that conveyed information on the functional distinctions between PMN and intermediate monocytes in their association with parameters of disease severity. Together, these findings are consistent with decreased endogenous SPM bioavailability in sepsis as a unifying mechanism for the dysregulated systemic inflammatory responses and their influence on clinical severity. In addition to uncovering insights into sepsis immunopathology in humans, these results provide methods for assessment of leukocytes in microliter quantities of peripheral blood, including measures of cell activation, SPM receptor expression, and functional responses to SPMs, that may ultimately be helpful in stratifying patients with sepsis and other critical illness in clinical research and care.

## Methods

### Sample collection and patient adjudication.

After obtaining informed consent from individuals or their surrogates, a volume of 1 mL of peripheral venous blood was obtained from a total of 31 patients with a possible diagnosis of sepsis and 8 healthy volunteers at the BWH. The clinical diagnosis was unknown to the investigators when the freshly isolated biospecimens were assayed on the day of collection. As part of the BWH Registry of Critical Illness, patients were assigned a diagnosis of sepsis for 18 subjects (based on the latest Sepsis-3 guidelines) (37) after the patient’s hospital course was reviewed by an independent panel of 3 critical care physicians. Peripheral blood samples were collected in EDTA-containing vacutainer tubes within 72 hours of admission to BWH medical intensive care unit (day 0) or admission to the hospital floor services who had positive blood cultures within 24 hours (day 0). In addition, peripheral blood from hospitalized patients were also obtained at days 3 and 7 after admission, when available. Freshly obtained biospecimens were processed within 1–2 hours of collection. Healthy individuals reported no medical history and took no over-the-counter medications in the 2 weeks prior to enrollment. Table 1 shows subject characteristics.

### Human leukocyte isolation.

Leukocytes were isolated from 50 μL of freshly obtained peripheral blood by using the inertial microfluidic separation for downstream assessment of cellular responses to RvD1 and RvD2 in disease and health. The design, assembly, and processing of the inertial microfluidics separation system (IDS) is described in published work elsewhere (7).

### Flow cytometry.

Using inertial microfluidic separation (7), 100,000 leukocytes were isolated from 50 μL of peripheral blood. Leukocytes were incubated with 5 μL of Fc-block (Human TruStain FcX, BioLegend, 422302) for 10 minutes at room temperature (RT). For DRV1 (GPR32; Abcam, ab79516) and DRV2 (GPR18; Abcam, ab76258) surface receptor staining, leukocytes were incubated with a primary antibody (1:50) to human proteins (anti-GPR32 or anti-GPR18, Abcam) for 20 minutes at RT. Cells were then washed with 1 mL of PBS (without Ca^2+^ and Mg^2+^). Leukocytes were incubated for 20 minutes at RT with following antibodies to human proteins after washing, with clones noted in parentheses: anti–CD45 PerCP (HI30), anti–CD66b Pacific blue (G10F5), anti–CD16 APC-Cy7 (3G8), and anti–CD14 PE-Cy7 (63D3) (all from BioLegend), as well as secondary antibody Alexa Fluor 546 goat anti–rabbit IgG (1:200) (Thermo Fisher Scientific, A11010). For ALX (FPR2) surface receptor staining, leukocytes were incubated for 20 minutes with the following antibodies to human proteins, with clones noted in parentheses: anti–CD45 PerCP (HI30), anti–CD66b Pacific blue (G10F5), anti–CD16 APC-Cy7 (3G8), and anti–CD14 PE-Cy7 (63D3) (all from BioLegend), as well as anti–hFPRL1/FPR2 PE (R&D systems, FAB3479P). After staining, cells were lysed and fixed with 2 mL of 1:4 dilution of Lyse/fix Buffer 5X (BD Phosflow) with distilled water for 15 minutes at RT. The gating strategy for the identification of PMN (FSC^+^SSC^+^CD45^+^CD66b^+^) and monocyte (FSC^+^SSC^–^CD45^+^CD66b^–^) subsets is demonstrated in Supplemental Figure 1. Data were acquired on a BD LSR Fortessa flow cytometer and were analyzed using FlowJo software version 10.1 (Tree star).

### PMN phagolysosome acidification assay.

PMN phagolysosome formation response to RvD1 and RvD2 in disease and health was assessed by using pHrodo red *E*. *coli* bioparticles conjugate. The preparation of pHrodo red *E*. *coli* bioparticles conjugate is described in previous work (7). Using the inertial microfluidic separation, 100,00 leukocytes were isolated from 50 μL of peripheral blood. Leukocytes were first incubated for 5 minutes at 37°C. Following incubation, cells were exposed to RvD1 (1, 10, or 100 nM), RvD2 (1, 10, or 100 nM), or vehicle (<0.01% v/v EtOH) for 15 minutes at 37°C. Leukocytes were then incubated for 15 minutes at 37°C after exposing cells with 25 μL of pHrodo. Cells were washed with 1 mL of PBS (without Ca^2+^ and Mg^2+^) and resuspended with 50 μL of PBS (without Ca^2+^ and Mg^2+^). A total of 2 μL of anti-human antibodies to human proteins were used to stain leukocytes, with clones noted in parentheses: anti–CD45 PerCP (HI30), anti–CD66b Pacific Blue (G10F5), anti–CD16 APC-Cy7 (3G8), and anti–CD14 PE-Cy7 (63D3) (all from BioLegend). After staining, the cells were lysed and fixed with 2 mL of 1:4 dilution of Lyse/fix Buffer 5X (BD Phosflow) with distilled water for 15 minutes at RT. Data were acquired on a BD LSR Fortessa flow cytometer and were analyzed using FlowJo software version 10.1 (Tree Star Inc.).

### PMN activation assessment in IDS.

PMN responses to RvD1 and RvD2 in disease and health were assessed by using the IDS. Using the MACSxpress Neutrophil Extraction Kit (Miltenyi Biotec) and a magnet (MACSxpress Separator, Miltenyi Biotec), PMN were isolated from 100 μL of freshly obtained peripheral blood. PMN were resuspended with PBS (without Ca^2+^ and Mg^2+^) after centrifugation at 200*g* for 5 minutes RT. PMN were then exposed to RvD1 (1, 10, or 100 nM), RvD2 (1, 10, or 100 nM), or vehicle (<0.01% v/v EtOH) for 15 minutes at 37°C. Following exposure, PMN were stimulated with PMA at 20 nM (Cayman Chemical) or vehicle (<0.01% v/v EtOH for 30 minutes at 37°C). After incubation, PMN were resuspended in PBS buffer (without Ca^2+^ and Mg^2+^) after centrifugation at 200*g* for 5 minutes at RT and introduced into the IDS chamber for processing. The design, processing, and image analysis of the IDS is described in published work elsewhere (7).

### Statistics.

Data in this study were shown as mean ± SEM, unless otherwise indicated. The assessment of statistical significance between groups was performed by 2-tailed Student’s *t* test or Mann-Whitney *U* test for data that were not normally distributed unless otherwise indicated. Correlations were tested using Pearson’s correlation coefficient (*r*). *P* < 0.05 was considered statistically significant. Statistics were performed using Prism 6.0 for Mac (GraphPad). For multivariate statistical analysis, PCA was performed for samples with complete data sets using R software. The mean *Z* scores were calculated using SPSS. The assessment of statistical significance between groups were performed by either 1-way ANOVA, 2-tailed Student’s *t* test, or Mann-Whitney *U* test for data that were not normally distributed unless otherwise indicated.

### Study approval.

Written informed consent was obtained after the approval by Partners Healthcare IRB under protocols 2002P000272, 2018P002489, 2008P000495, and 2017P000367.

## Author contributions

BDL, JV, JH, and RMB conceived the study. BJ, DHL, MGD, REEA, RMB, JH, JV, and BDL designed the experiments and interpreted the results. BJ, DHL, REEA, JV, and BDL performed and analyzed the functional biological experiments, and HJ and JH designed and performed experiments using the inertial microfluidic system. MPV and RMB provided blood samples and clinical data from patients with sepsis. The manuscript was written by BJ, DHL, HJ, MGD, JN, REA, RMB, JH, JV, and BDL.

## Supplementary Material

Supplemental data

## Figures and Tables

**Table 1:**
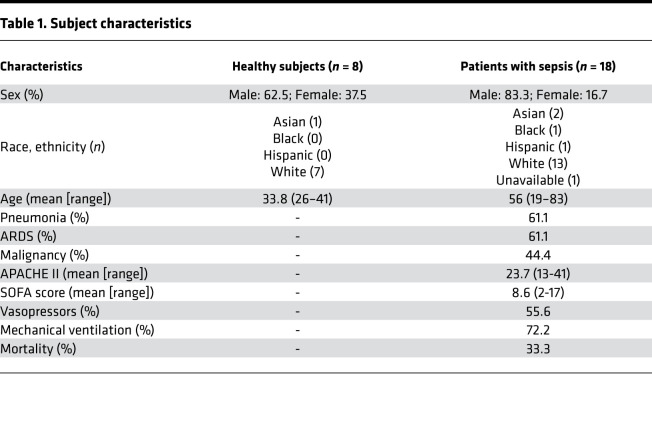
Subject characteristics

## Version 1 06/24/2021

In-Press Preview

## Version 2 08/09/2021

Electronic publication

## References

[1] Rhee C (2017). Incidence and trends of sepsis in US hospitals using clinical vs claims data, 2009-2014. JAMA.

[2] Seymour CW (2016). Assessment of clinical criteria for sepsis: for the third international consensus definitions for sepsis and septic shock (Sepsis-3). JAMA.

[3] Singer M (2016). The third international consensus definitions for sepsis and septic shock (Sepsis-3). JAMA.

[4] Delano MJ, Ward PA (2016). Sepsis-induced immune dysfunction: can immune therapies reduce mortality?. J Clin Invest.

[5] Pierrakos C, Vincent JL (2010). Sepsis biomarkers: a review. Crit Care.

[6] Basil MC, Levy BD (2016). Specialized pro-resolving mediators: endogenous regulators of infection and inflammation. Nat Rev Immunol.

[7] Jundi B (2019). Leukocyte function assessed via serial microlitre sampling of peripheral blood from sepsis patients correlates with disease severity. Nat Biomed Eng.

[8] Padovan MG, Norling LV (2020). Pro-resolving lipid mediators in sepsis and critical illness. Curr Opin Clin Nutr Metab Care.

[9] Dalli J (2017). Human sepsis eicosanoid and proresolving lipid mediator temporal profiles: correlations with survival and clinical outcomes. Crit Care Med.

[10] Serhan CN (2014). Lipid mediators in the resolution of inflammation. Cold Spring Harb Perspect Biol.

[11] Serhan CN, Levy BD (2018). Resolvins in inflammation: emergence of the pro-resolving superfamily of mediators. J Clin Invest.

[12] Krishnamoorthy N (2018). Specialized proresolving mediators in innate and adaptive immune responses in airway diseases. Physiol Rev.

[13] Spite M (2009). Resolvin D2 is a potent regulator of leukocytes and controls microbial sepsis. Nature.

[14] Tejera P (2020). Plasma levels of proresolving and prophlogistic lipid mediators: association with severity of respiratory failure and mortality in acute respiratory distress syndrome. Crit Care Explor.

[15] Lee CR, Zeldin DC (2015). Resolvin infectious inflammation by targeting the host response. N Engl J Med.

[16] Chiang N (2012). Infection regulates pro-resolving mediators that lower antibiotic requirements. Nature.

[17] Summers C (2019). Chasing the “Holy Grail”: modulating neutrophils in inflammatory lung disease. Am J Respir Crit Care Med.

[18] Hsiao HM (2014). Resolvin D1 attenuates polyinosinic-polycytidylic acid-induced inflammatory signaling in human airway epithelial cells via TAK1. J Immunol.

[19] Hsiao HM (2013). A novel anti-inflammatory and pro-resolving role for resolvin D1 in acute cigarette smoke-induced lung inflammation. PLoS One.

[20] Chen F (2014). Resolvin D1 improves survival in experimental sepsis through reducing bacterial load and preventing excessive activation of inflammatory response. Eur J Clin Microbiol Infect Dis.

[21] Wang M (2020). Resolvin D1 protects against sepsis-induced cardiac injury in mice. Biofactors.

[22] Summers C (2010). Neutrophil kinetics in health and disease. Trends Immunol.

[23] Chiang N (2017). Novel resolvin D2 receptor axis in infectious inflammation. J Immunol.

[24] Kurihara T (2013). Resolvin D2 restores neutrophil directionality and improves survival after burns. FASEB J.

[25] Abdulnour RE (2016). Aspirin-triggered resolvin D1 is produced during self-resolving gram-negative bacterial pneumonia and regulates host immune responses for the resolution of lung inflammation. Mucosal Immunol.

[26] Poulsen RC (2008). Identification of inflammatory and proresolving lipid mediators in bone marrow and their lipidomic profiles with ovariectomy and omega-3 intake. Am J Hematol.

[27] Stenke L (1994). Leukotrienes and lipoxins--new potential performers in the regulation of human myelopoiesis. Leuk Res.

[28] Ziegler-Heitbrock L (2014). Monocyte subsets in man and other species. Cell Immunol.

[29] Reyes M (2020). An immune-cell signature of bacterial sepsis. Nat Med.

[30] Abdulnour RE (2018). Early intravascular events are associated with development of acute respiratory distress syndrome. A substudy of the LIPS-A clinical trial. Am J Respir Crit Care Med.

[31] Krishnamoorthy S (2010). Resolvin D1 binds human phagocytes with evidence for proresolving receptors. Proc Natl Acad Sci U S A.

[32] Chiang N (2015). Identification of resolvin D2 receptor mediating resolution of infections and organ protection. J Exp Med.

[33] Ellett F (2018). Diagnosis of sepsis from a drop of blood by measurement of spontaneous neutrophil motility in a microfluidic assay. Nat Biomed Eng.

[34] Hassan U (2017). A point-of-care microfluidic biochip for quantification of CD64 expression from whole blood for sepsis stratification. Nat Commun.

[35] Kemmler MS (2014). Biochip point-of-care device for sepsis diagnostics. Sens Actuators B Chem.

[36] Reddy B (2018). Point-of-care sensors for the management of sepsis. Nat Biomed Eng.

[37] Singer M (2016). The Third International Consensus Definitions for Sepsis and Septic Shock (Sepsis-3). JAMA.

