# Supplementary material for: Inflammation resolution circuits are uncoupled in acute sepsis and correlate with clinical severity
Source: JCI Insight. 2021 Aug 9;6(15):e148866. doi: 10.1172/jci.insight.148866 (PMC8410067; doi:10.1172/jci.insight.148866)
Supplement: Supplemental data [file jciinsight-6-148866-s008.pdf]

Inflammation resolution circuits are uncoupled in acute sepsis and correlate with clinical severity

Bakr Jundi[1], Do-Hyun Lee[2], Hyungkook Jeon[2], Melody G. Duvall[1], Julie Nijmeh[1], Raja-Elie E. Abdulnour[1], Mayra Pinilla-Vera [1], Rebecca M. Baron[1], Jongyoon Han[2], Joel Voldman[2], Bruce D. Levy[1*]

ONLINE DATA SUPPLEMENT

Supplementary Figure 1. Flow cytometry gating strategy for identification of PMN and monocyte subsets isolated from peripheral blood. From 50 μL of collected peripheral blood, leukocytes were isolated using the closed-loop operation of spiral microfluidics system (see methods). On the flow cytometry contour plots, PMNs were identified by $FSC^+SSC^+CD45^+CD66b^+$ and its subsets were identified by CD16 and CD66b surface expression. Monocytes were identified by $FSC^+SSC^-CD45^+CD66b^-$ and its subsets were identified by CD16 and CD14 surface expression. CM, classical monocytes, IM, intermediate monocytes, NCM, non-classical monocytes.

Supplementary Figure 2. DRV1, ALX and DRV2 receptor expression during hospitalization stay and PMN responses to exogenous RvD1 and RvD2. From 50 μL of collected peripheral blood, leukocytes were isolated using the closed-loop operation of spiral microfluidics system (see methods). **(A)** The mean fluorescent intensity (MFI) of surface expression of DRV1, ALX and DRV2 on all PMN subsets was determined in sepsis patients at day 0 and during their 7-day hospitalization. **(B)** $CD16^{bright}$ PMNs from healthy subjects were exposed to a pHrodo-labelled *E. coli* bioparticles for 15 min at 37°C and 4°C to determine the gating strategy. **(C)** The frequency of $pHrodo^+$ $CD16^{bright}$ PMNs after exposure to vehicle (<0.01 EtOH, circle), RvD1 (100 nM, square), or RvD2 (100 nM, triangle) in sepsis at day 0 and during their 7-day hospitalization. **(D)** The absolute increase of $pHrodo^+$ in all subsets of PMN with exogenous RvD1 and RvD2 as measured by SPM sepsis – sepsis vehicle. Values are expressed as the mean +/- s.e.m. n = 3 healthy subjects, n = 18 patients with sepsis.

Supplementary Figure 3. Effect of RvD1 and RvD2 on response of PMN from healthy and sepsis patients to PMA stimulation. PMN were isolated from 100 μL of peripheral blood using the magnetic activated cell sorting (MACS) kit (see methods), then first incubated with vehicle (<0.01 v/v EtOH), RvD1 (100 nM), or RvD2 (100 nM) for 15 minutes, followed by PMA (red) or vehicle (black) stimulation for 30 minutes. Their IDPs were measured at 7 MHz frequency. **(A)** Representative histogram plots of IDP distribution and **(B)** measurement of median IDP in response to RvD1 and RvD2 in PMA-stimulated (red) and non-stimulated (black) PMNs isolated from healthy subjects. The dashed lines in A represent the median IDP. **(C)** Box and whisker plots (median, 25th and 75th percentiles) of the Δ_{med}IDP (calculated by Median IDP (non-stimulated) – Median IDP (stimulated)) in sepsis and health. RvD1 and RvD2 dose-response curves of median IDP of non-stimulated PMN **(D)** and Δ_{med}IDP **(E)** from healthy subjects. n=3-4 healthy subjects and n=4 sepsis patients.

Supplementary Figure 4. Response of various monocyte subsets in sepsis to RvD1 and RvD2. From 50 μL of peripheral blood, monocytes were isolated using the closed-loop operation of spiral microfluidics system (see methods). **(A)** Level of surface receptor expression of DRV1, ALX and DRV2 expressed as MFI on all monocyte subsets (CM, IM, NCM) were determined in sepsis patients at day 0 and throughout their hospitalization. **(B)** Frequency of pHrodo^{+} classical monocytes in sepsis (crimson) and health (dark gray) after incubation with exogenous RvD1 (100 nM), RvD2 (100 nM), or vehicle (<0.01% v/v EtOH) for 15 min at 37°C. **(C)** Absolute increase in frequency of pHrodo^{+} of all monocyte subsets (CM, IM, NCM) in sepsis patients. **(D)** Dose-response curve of the frequency of pHrodo^{+} classical monocytes to varying concentrations of RvD1 (circle, crimson), RvD2 (square, crimson), or vehicle (mean value, dashed gray line).

*P<0.05 intermediate vs. non-classical monocyte in sepsis patients by paired, two-tailed t-test. Values are expressed as the mean +/- s.e.m. n = 17 patients with sepsis.

Supplementary Figure 5. Differential expression of DRV1, ALX and DRV2 receptors and functional responses in sepsis are counter regulated by RvD1 and RvD2. Two-dimensional score and loading plots from multivariate principal component analysis **(A & C)** and mean Z-score **(B & D)** were performed for DRV1, ALX, and DRV2 receptor expression on leukocytes, and leukocyte activation and function as indicated by Δ_{med}IDP and percentage of pHrodo^{+} CD16^{bright} PMN, and pHrodo^{+} classical monocytes (CM). Healthy subjects are indicated in red, sepsis patients treated with vehicle control are in green, addition of RvD1 and RvD2 is indicated in blue and aquamarine, respectively. **(A & B)** PCA and mean Z-score for SPM receptor expression were from n=4 healthy subjects and n = 10 sepsis patients. **(C & D)** PCA and mean Z-score for leukocyte activation and function parameters were from n=4 healthy subjects and n = 4 sepsis patients; as complete functional data were only available for these 4 patients with sepsis. The mean Z-score was derived from individual variable Z-scores in a given subject. See Fig. 4 for PCA analysis for subjects with complete data set of receptors, function, and activation. Values are expressed as the mean +/- s.e.m. *P<0.05 health vs. sepsis by unpaired, two-tailed t-test. §P<0.05 Kruskal-Wallis test, followed by Dunn's test for multiple comparisons.

Supplementary Figure 6. Correlation between absolute increase of frequency of pHrodo^{+} CD16^{bright} PMN with exogenous RvD1 and sepsis clinical severity. (A) The relationship between clinical severity indicators (SOFA, APACHE II, status of mechanical ventilation, and mortality outcome) and absolute increase of pHrodo^{+} CD16^{bright} PMN with exogenous RvD1. n =

11 sepsis patients. The Pearson correlation r value and significance are noted, and regression lines are shown.

Supplementary Figure 7. Correlation between absolute increase of frequency of pHrodo^{+} CD16^{bright} PMN with exogenous RvD2 and sepsis clinical severity. (A) The relationship between clinical severity indicators (SOFA, APACHE II, status of mechanical ventilation, and mortality outcome) and absolute increase of pHrodo^{+} CD16^{bright} PMN with exogenous RvD2. n = 10 patients with sepsis. The Pearson correlation r value and significance are noted, and regression lines are shown.

Supplemental Figure 1

A

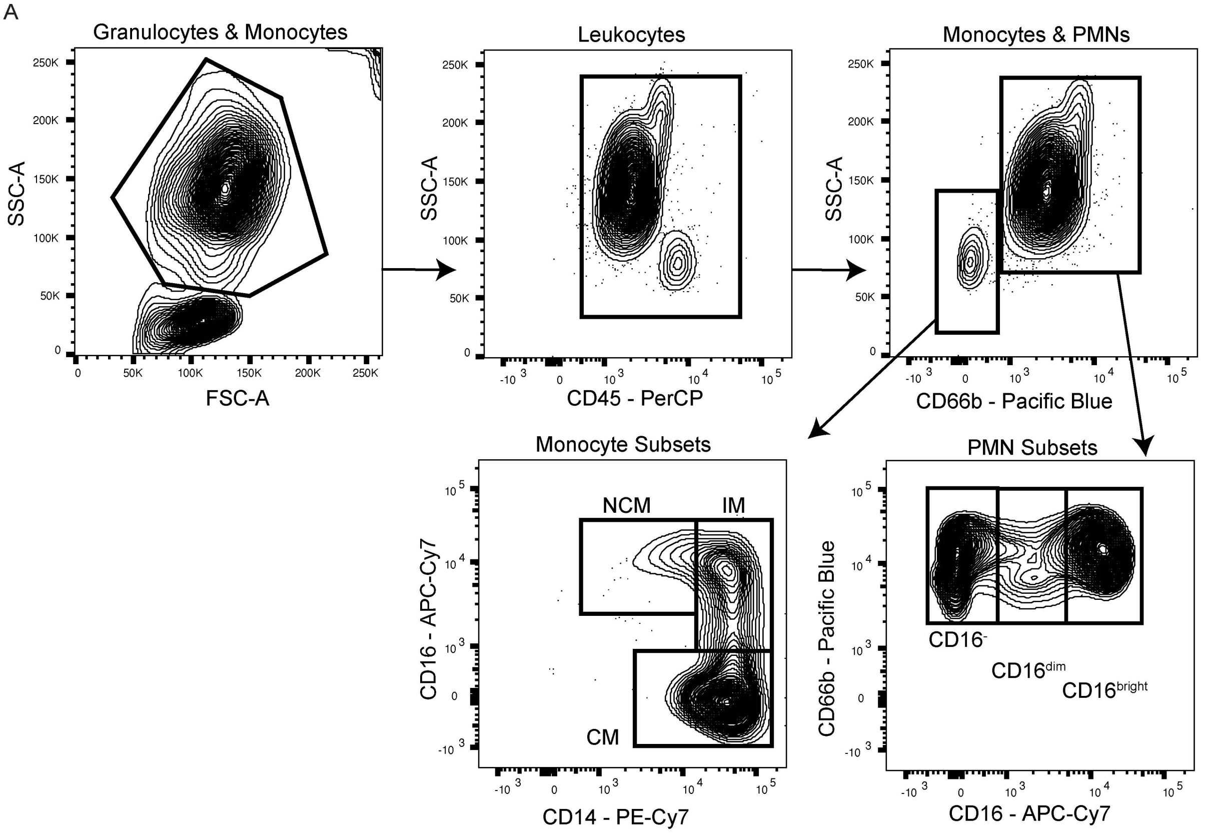

Supplemental Figure 1. Flow cytometry gating strategy for identification of PMN and monocyte subsets isolated from peripheral blood. From 50 μL of collected peripheral blood, leukocytes were isolated using the closed-loop operation of spiral microfluidics system (see methods). **(A)** On the flow cytometry contour plots, PMNs were identified by $FSC^+SSC^+CD45^+CD66b^+$ and its subsets were identified by CD16 and CD66b surface expression. Monocytes were identified by $FSC^+SSC^-CD45^+CD66b^-$ and its subsets were identified by CD16 and CD14 surface expression. CM, classical monocytes, IM, intermediate monocytes, NCM, non-classical monocytes.

Supplemental Figure 2

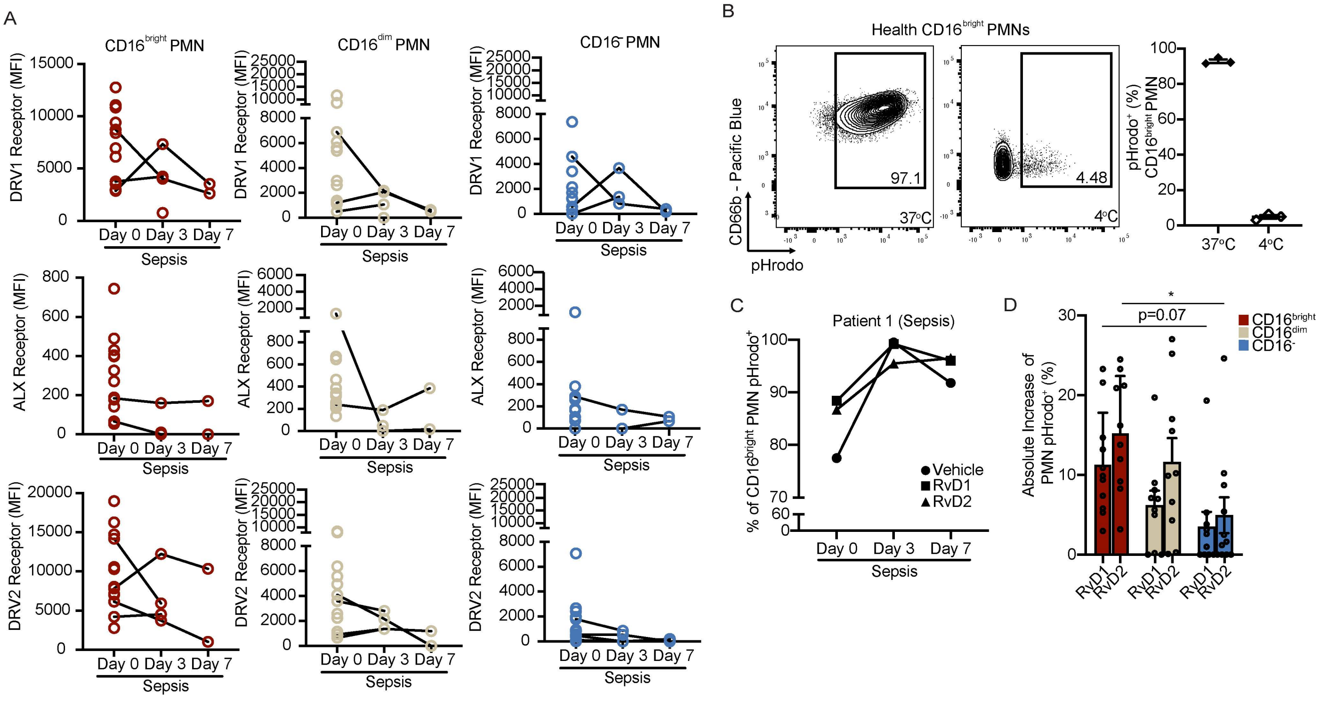

Supplemental Figure 2. DRV1, ALX and DRV2 receptor expression during hospitalization stay and PMN responses to exogenous RvD1 and RvD2. From 50 µL of collected peripheral blood, leukocytes were isolated using the closed-loop operation of spiral microfluidics system (see methods). **(A)** The mean fluorescent intensity (MFI) of surface expression of DRV1 (n=12), ALX (n=13) and DRV2 (n=13) on all PMN subsets was determined in sepsis patients at day 0 and during their 7-day hospitalization. **(B)** CD16^{bright} PMNs from healthy subjects (n=3) were exposed to a pHrodo-labelled E. coli bioparticles for 15 min at 37°C and 4°C to determine the gating strategy. **(C)** The frequency of pHrodo^{+} CD16^{bright} PMNs after exposure to vehicle (<0.01 EtOH, circle), RvD1 (100 nM, square), or RvD2 (100 nM, triangle) in sepsis at day 0 and during their 7-day hospitalization. **(D)** The absolute increase of pHrodo^{+} in all subsets of PMN with exogenous RvD1 (n=11) and RvD2 (n=10) as measured by SPM sepsis – sepsis vehicle. Values are expressed as the mean +/- s.e.m.*P<0.05 for absolute increase of PMN subsets pHrodo^{+} of RvD2 by one-way analysis of variance (ANOVA).

Supplemental Figure 3

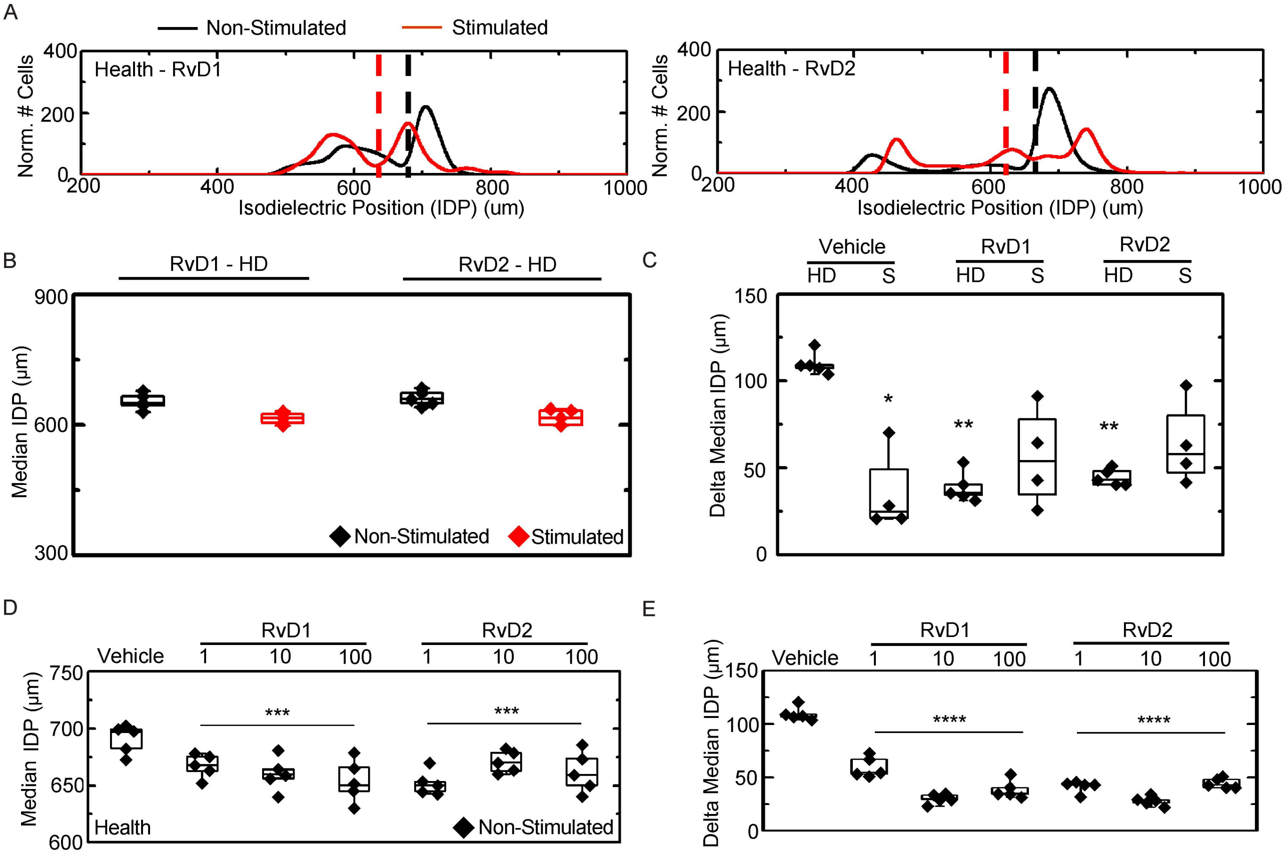

Supplemental Figure 3. Effect of RvD1 and RvD2 on response of PMN from healthy and sepsis patients to PMA stimulation. PMN were isolated from 100 µL of peripheral blood using the magnetic activated cell sorting (MACS) kit (see methods), then first incubated with vehicle (<0.01 v/v EtOH), RvD1 (100 nM), or RvD2 (100 nM) for 15 minutes, followed by PMA (red) or vehicle (black) stimulation for 30 minutes. Their IDPs were measured at 7 MHz frequency. **(A)** Representative histogram plots of IDP distribution and **(B)** measurement of median IDP in response to RvD1 and RvD2 in PMA-stimulated (red) and non-stimulated (black) PMNs isolated from healthy subjects. The dashed lines in A represent the median IDP. **(C)** Box and whisker plots (median, 25th and 75th percentiles) of the Δ_{med}IDP (calculated by Median IDP (non-stimulated) – Median IDP (stimulated)) in sepsis and health. RvD1 and RvD2 dose-response curves of median IDP of non-stimulated PMN **(D)** and Δ_{med}IDP **(E)** from healthy subjects. *P<0.05 for Δ_{med}IDP of vehicle exposed PMN in health vs sepsis by unpaired, two-tailed t-test. **P<0.05 for Δ_{med}IDP of PMNs in health exposed to RvD1 & RvD2 vs vehicle by paired, two-tailed t-test. ***P<0.05 for concentration-response curve of the median IDP of non-stimulated PMN from healthy individuals with exogenous vehicle, RvD1, and RvD2 by one-way ANOVA. ****P<0.05 for concentration-response curve of the Δ_{med}IDP of non-stimulated PMN from healthy individuals with exogenous vehicle, RvD1, and RvD2 by one-way ANOVA. n=5 healthy subjects and n=4 sepsis patients.

Supplemental Figure 4

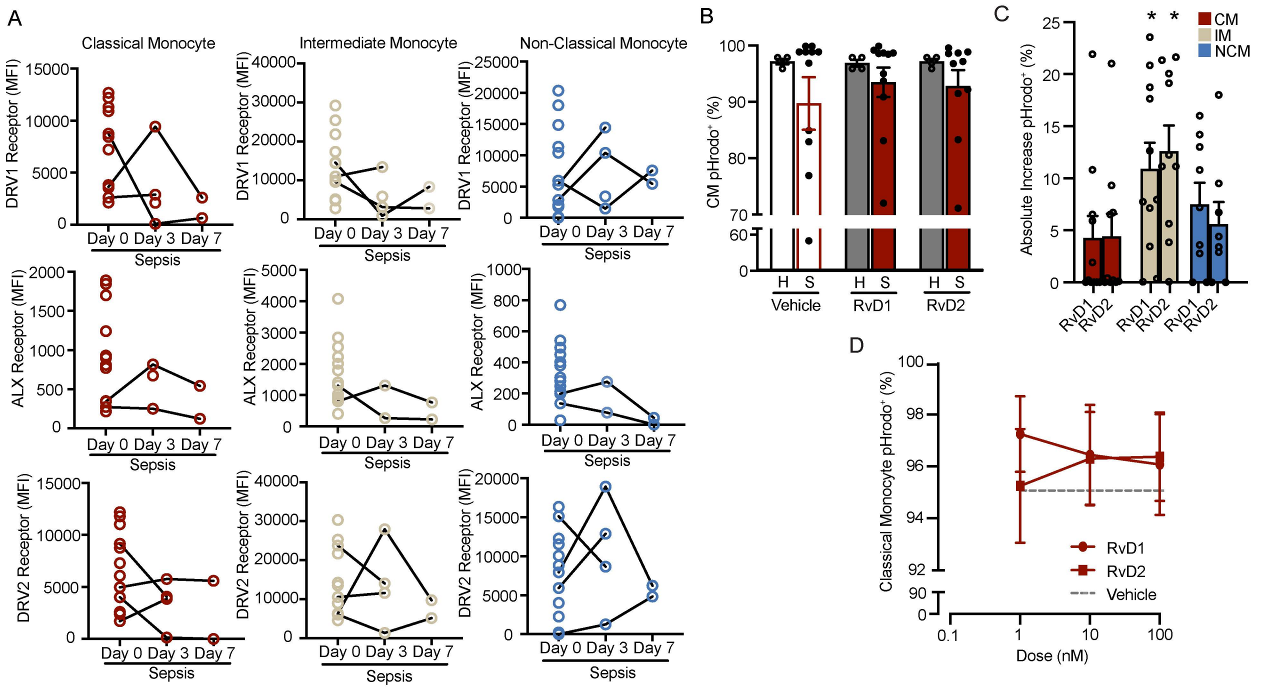

Supplemental Figure 4. Response of various monocyte subsets in sepsis to RvD1 and RvD2. From 50 µL of peripheral blood, monocytes were isolated using the closed-loop operation of spiral microfluidics system (see methods). **(A)** Level of surface receptor expression of DRV1 (n=12), ALX (n=13) and DRV2 (n=13) expressed as MFI on all monocyte subsets (CM, IM, NCM) were determined in sepsis patients at day 0 and throughout their hospitalization. **(B)** Frequency of pHrodo⁺ classical monocytes in sepsis (crimson, n=11) and health (dark gray, n=4) after incubation with exogenous RvD1 (100 nM), RvD2 (100 nM), or vehicle (<0.01% v/v EtOH) for 15 min at 37°C. **(C)** Absolute increase in frequency of pHrodo⁺ of all monocyte subsets (CM, IM, NCM) in sepsis patients (n=10-11). **(D)** Dose-response curve of the frequency of pHrodo⁺ classical monocytes to varying concentrations of RvD1 (circle, crimson), RvD2 (square, crimson), or vehicle (mean value, dashed gray line, n=6). *P<0.05 intermediate vs. non-classical monocyte in sepsis patients by paired, two-tailed t-test. Values are expressed as the mean +/- s.e.m.

Supplemental Figure 5

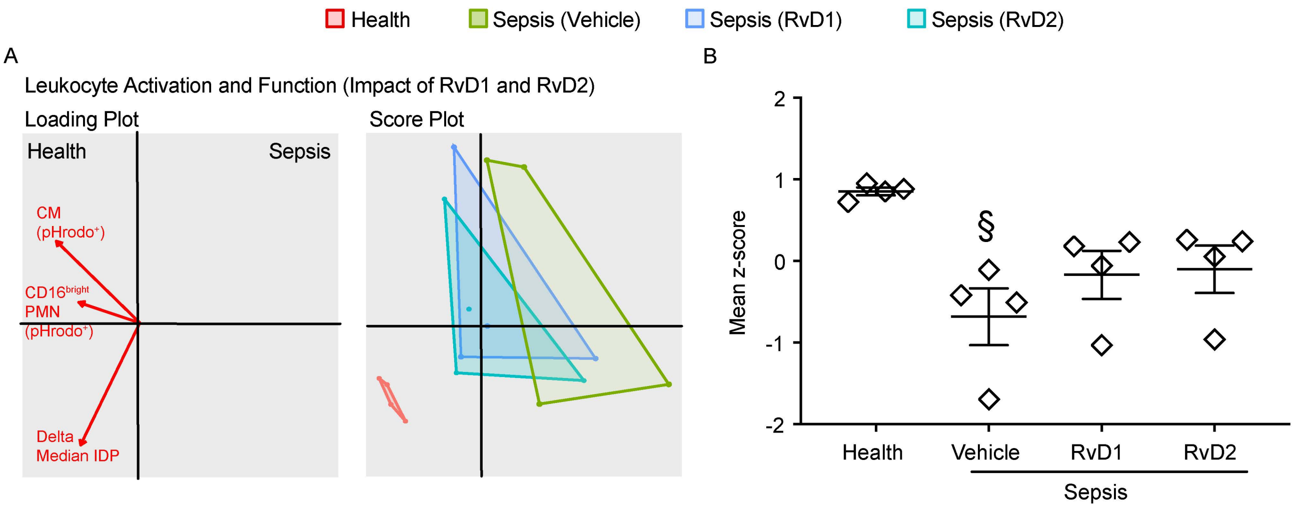

Supplemental Figure 5. Differential expression of DRV1, ALX and DRV2 receptors and functional responses in sepsis are counter regulated by RvD1 and RvD2. Two-dimensional score and loading plots from multivariate principal component analysis **(A)** and mean Z-score **(B)** were performed for leukocyte activation and function as indicated by Δ_{med}IDP and percentage of pHrodo^{+} CD16^{bright} PMN, and pHrodo^{+} classical monocytes (CM). Healthy subjects are indicated in red, sepsis patients treated with vehicle control are in green, addition of RvD1 and RvD2 is indicated in blue and aquamarine, respectively. **(A & B)** PCA and mean Z-score for leukocyte activation and function parameters were from n=4 healthy subjects and n = 4 sepsis patients; as complete functional data were only available for these 4 patients with sepsis. The mean Z-score was derived from individual variable Z-scores in a given subject. See Figure. 4 for PCA analysis for subjects with complete data set of receptors, function, and activation. Values are expressed as the mean +/- s.e.m. P<0.05 Kruskal-Wallis test, followed by Dunn’s test for multiple comparisons.

Supplemental Figure 6

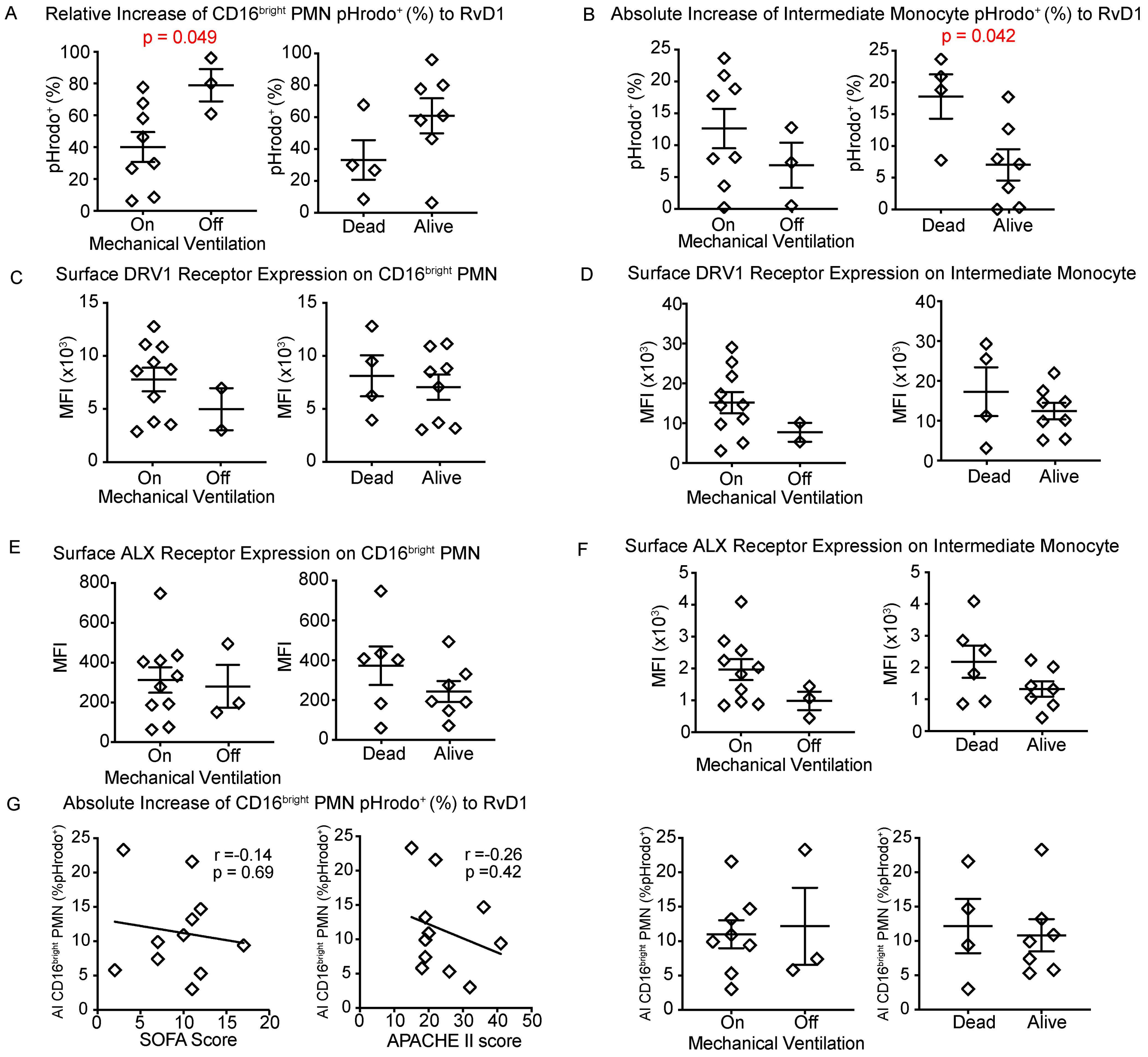

Supplemental Figure 6. Relationship between leukocyte responses to RvD1 and sepsis clinical severity. The correlation between severity indicators (status of mechanical ventilation and mortality outcome) and **(A)** relative increase of $CD16^{bright}$ PMN $pHrodo^{+}$ to RvD1 (n=11), **(B)** absolute increase of intermediate monocyte $pHrodo^{+}$ to RvD1 (n=11), and surface expression of DRV1 **(C,D)** (n=12) and ALX **(E,F)** (n=13) on $CD16^{bright}$ PMN and intermediate monocyte were determined. The relationship between the **(G)** absolute increase of $CD16^{bright}$ PMN $pHrodo^{+}$ to RvD1 (n=11) and clinical severity indcators (SOFA, APACHE II, status of mechanical ventilation, and mortality outcome) was determined. The Pearson correlation r value and significance are noted, and regression lines are shown. Values are expressed as the mean +/- s.e.m.

Supplemental Figure 7

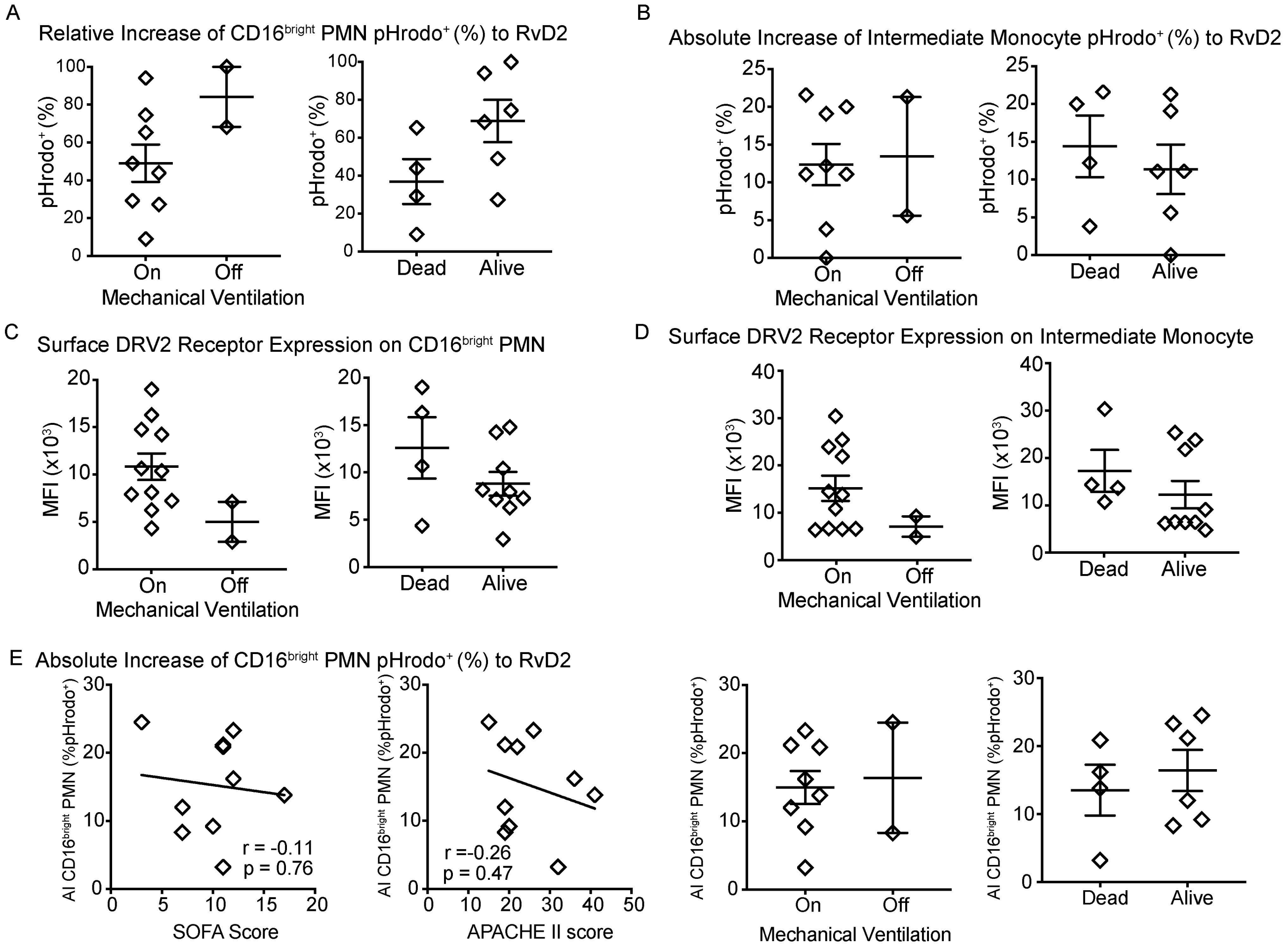

Supplemental Figure 7. Relationship between leukocyte responses to RvD2 and sepsis clinical severity. The correlation between severity indicators (status of mechanical ventilation and mortality outcome) and **(A)** relative increase of $CD16^{bright}$ PMN $pHrodo^{+}$ to RvD2 (n=10), **(B)** absolute increase of intermediate monocyte $pHrodo^{+}$ to RvD2 (n=10), and surface expression of DRV2 **(C,D)** (n=12) on $CD16^{bright}$ PMN and intermediate monocyte were determined. The relationship between the **(E)** absolute increase of $CD16^{bright}$ PMN $pHrodo^{+}$ to RvD2 (n=11) and clinical severity indcators (SOFA, APACHE II, status of mechanical ventilation, and mortality outcome) was determined. The Pearson correlation r value and significance are noted, and regression lines are shown. Values are expressed as the mean +/- s.e.m.
